# A generalizability analysis framework for bias resilience in convolution neural network and transformer models for diabetic retinopathy screening

**DOI:** 10.1371/journal.pone.0351225

**Published:** 2026-08-20

**Authors:** Majida Kazmi, Bisma Imran, Saad A. Qazi, M. A. Rehman Siddiqui

**Affiliations:** 1 Faculty of Electrical and Computer Engineering, NED University of Engineering & Technology, Karachi, Pakistan; 2 Neurocomputation Lab, National Centre of Artificial Intelligence, NED University of Engineering & Technology, Karachi, Pakistan; 3 Department of Surgery, Agha Khan University Hospital, Karachi, Pakistan; University of Manitoba, CANADA

## Abstract

Automated diabetic retinopathy (DR) screening has seen extensive research progress, yet its clinical adoption remains limited, largely due to insufficient attention to model generalizability across diverse populations and imaging conditions. Existing studies often overlook the role of dataset biases, arising from demographic, acquisition, and preprocessing variations that undermine robustness in real-world settings. To address this critical gap, we propose a comprehensive generalizability analysis framework that systematically categorizes dataset biases, evaluates their impact on performance, and introduces robust inter-dataset comparison metrics. The framework is metric-agnostic and extensible to multi-class severity grading, with stage-wise BAG formulations introduced to support more detailed clinical evaluation. Using one primary (EyePACS) and nine secondary datasets, we assessed the generalizability of two distinct architectures: a convolutional neural network (MobileNetV2) and a transformer-based model (CvT). Performance was evaluated through intra-group measures (accuracy, sensitivity, specificity, and AUC) and a newly proposed Bias-Adjusted Generalization (BAG) index designed to quantify resilience to bias-induced domain shifts. Results demonstrate that CvT consistently outperformed MobileNetV2, achieving an average accuracy of 87% and a BAG index of 0.94, compared to MobileNetV2’s 78% accuracy and BAG index of 0.91. Gradient-based explainability visualizations further confirm that both models attend to clinically relevant retinal regions, with architectural differences in activation patterns consistent with their respective generalizability profiles. These findings highlight the inherent bias resilience of transformer architectures, while highlighting their computational demands as a barrier to deployment in low-resource environments. Importantly, the study suggests that integrating transformer-inspired mechanisms into lightweight CNNs could yield clinically scalable models with both efficiency and robustness. Our framework offers a standardized approach for bias-aware evaluation, providing actionable insights for developing equitable, generalizable, and resource-adaptable AI solutions for DR screening.

## Introduction

**Fig 1:**
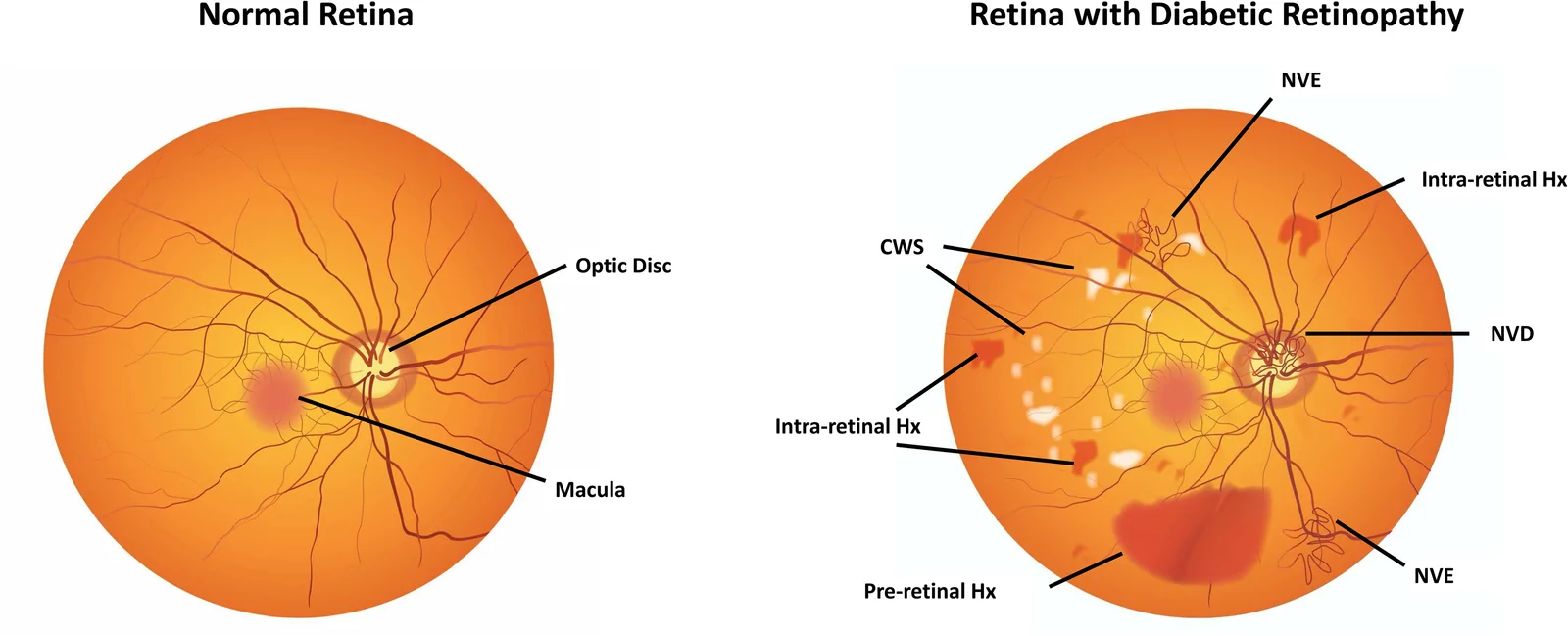
Comparison of a normal retina with one with diabetic retinopathy.

**Fig 2:**
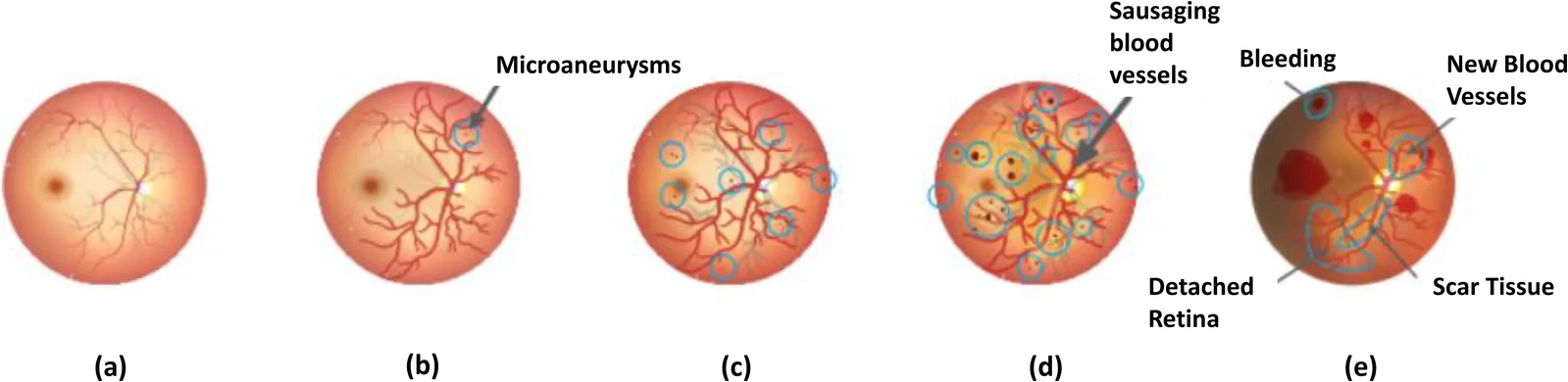
Different stages of diabetic retinopathy (a) Normal (b) Mild (c) Moderate (d) Severe (e) Proliferative [4].

Diabetes Mellitus (DM), or Diabetes, is a condition in which a person has increased blood sugar levels over an extended period. Diabetic Retinopathy (DR), a complication of DM, can potentially lead to permanent blindness. According to the latest IDF Diabetes Atlas (2025) report, one in nine adults, approximately 589 million are currently living with diabetes, and this is projected to increase to one in eight adults, reaching approximately 853 million by 2050 [1]. According to other estimates [2], the global prevalence of DR is approximately 103 million individuals and is projected to increase to 161 million individuals by 2045. However, this condition and the likelihood of complete vision loss can be prevented in up to 95% of DR patients with early diagnosis [3]. The severity of DR is characterized by several abnormalities such as intra-retinal and pre-retinal hemorrhages (Hx), neovascularization elsewhere (NVE), neovascularization of the disc (NVD), and cotton wool spots (CWS), as highlighted in Fig 1. Based on these abnormalities, DR can be divided into 5 stages as illustrated in Fig 2.

Traditional methods for DR diagnosis faced problems especially in low- and middle-income countries (LMICs); as it required the presence of expert ophthalmologists, which is quite a challenge due to the low ratio of ophthalmologists to DR affected population.

Artificial Intelligence (AI) has shown remarkable success across various domains and is increasingly being integrated into healthcare solutions. However, the effectiveness of AI in clinical settings depends largely on the generalizability of the models, that is, their ability to perform reliably under diverse conditions and across different populations. A key challenge to achieving generalizability lies in the presence of dataset biases during model training. These biases arise from variations in imaging conditions, devices, populations, or dataset characteristics, and they can significantly influence model performance.

Importantly, such biases may alter image features of the retina that are unrelated to the disease of interest. Since AI models typically process entire retinal images, they may learn through biased features rather than pay attention to the disease-relevant features results in reducing the robustness and thus clinical applicability. Therefore, two aspects become critical: (i) systematically identifying and understanding the sources and types of dataset biases, and (ii) selecting models that are capable of emphasizing clinically meaningful features while ignoring irrelevant variations.

Both CNN-based and transformer-based architectures are increasingly used in clinical diagnostic tasks across medical imaging. Transformer-based models have demonstrated competitive or superior performance compared to traditional CNNs in certain settings, such as breast ultrasound image classification [5], highlighting the importance of systematic architectural comparison. However, despite these advances, the generalizability of different architectural paradigms across diverse datasets remains insufficiently explored in DR screening.

Many studies have been conducted on automated DR detection, however most of them focus on enhancing screening performance for a particular dataset, often ignoring the impact of dataset-specific biases on real-world robustness. Despite mentioned from time to time, cross-dataset validation is rarely carried out in a methodical or bias-aware manner. Most importantly, no standardized framework exists at this time that directly links the sources of dataset bias to measurable degradation in model generalization ability.

To address this challenge, a systematic framework is required, one that first categorizes dataset biases, then evaluates their impact on model performance, and finally applies robust metrics to analyze generalizability. The objective of this study is to develop and adapt such a standardized framework. Specifically, we categorize different biases present in retinal imaging datasets and correlate them with model performance. To validate our approach, we selected two architecturally distinct models and evaluated them across multiple datasets. This comparison highlights how architectural design influences a model’s ability to handle biases, thereby offering insights into the development of more generalizable AI solutions for automated DR screening. Building on the identified gap, the main contributions of this work are as follows:

- **Categorization and correlation of data biases:** We performed an in-depth analysis for the source-wise categorization of data biases at different stages of the dataset creation phase and correlated them with the generalizability of the model.
- **Comprehensive framework for generalizability analysis:** We proposed a comprehensive framework of its kind, for generalizability analysis. Starting with the study of data biases and their effect on AI model’s performance, we then chose datasets that are different from each other and can potentially lead to data bias. These datasets were used to evaluate the inherent generalizability of a CNN-based and Transformer-based model. The generalizability analysis was carried out using different performance metrics for both intra-group and inter-group analyses.
- **Introduction of the Bias-Adjusted Generalization (BAG) index:** To accurately capture inter-group performance differences while accounting for dataset biases, we introduce a metric called BAG index. Rather than merely calculating the accuracy drop on the test set compared to the training set, BAG index quantifies model generalization by incorporating both the normalized performance drops and the normalized magnitude of key biases between datasets. This provides a balanced, normalized score that reflects the model’s ability to maintain reliable performance across biased datasets.

**Fig 3:**
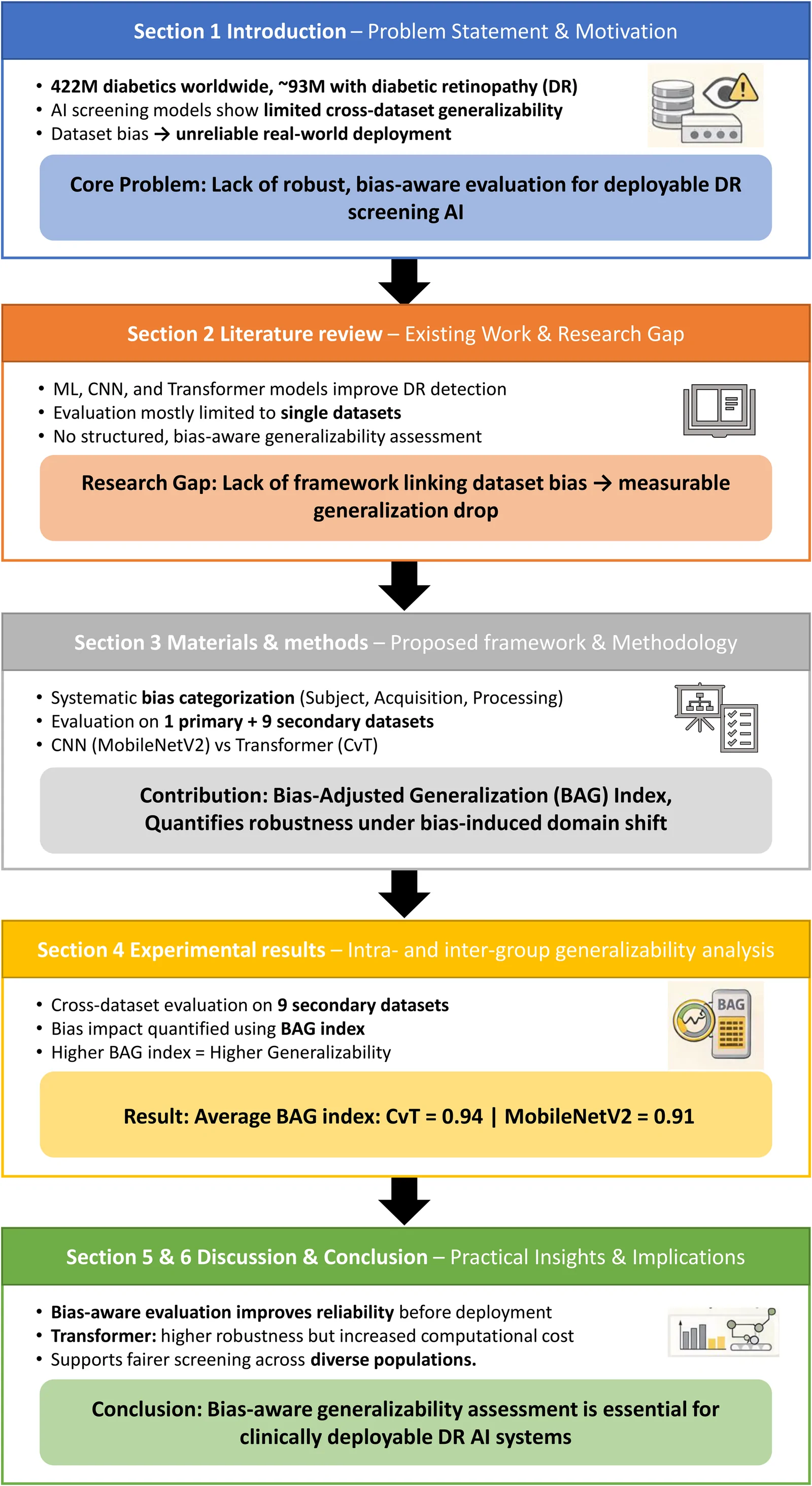
The graphical abstract of the proposed study.

Fig 3 presents a graphical abstract that provides a clear visual summary of the manuscript’s structure and the study’s logical flow. Its blocks correspond to different stages of the study, starting with problem identification and research gap definition, and progressing through framework development, experimental results, and practical conclusions.

The rest of the paper is organized as follows. The Literature review section provides an overview of current machine learning (ML) and deep learning (DL) techniques for DR screening, with an emphasis on generalizability constraints. The Materials and methods introduces bias-aware generalizability analysis framework, including information about dataset selection, bias categorization, model architecture and evaluation metrics. The Experimental results section present both intra- and inter-dataset evaluations, including analysis using the proposed BAG index. Finally, Discussion and future directions and Conclusion sections interpret the findings, outline practical implications, and identify future research directions.

## Literature review

In this section, previous work on DR screening is reviewed, with a focus on model architectures and the degree to which dataset bias and generalizability have been addressed. With the increasing prevalence of DR, the research community has been looking for the most efficient and accurate techniques for its early detection. Over the years, both machine learning (ML) [6–8] and DL-based [9–18] techniques have been used for this purpose, as summarized in Table 1. In study [6], Minhaz et al. evaluated several ML algorithms for DR prediction and concluded that of these, the LR classifier showed the highest performance with 75% accuracy and 83% receiver operating curve (ROC) value. In study [7], multiple ML classifiers were evaluated for automated DR detection and the results showed that the Ranger Random Forest (RRF) outperformed other classifiers by achieving an accuracy of 86%. G. T. Reddy et al. in [8] used an ensemble learning approach for classifying DR. An ensemble-based ML model was created using different ML models, and its performance was compared with individual classifiers. The results revealed that ensemble-based ML model outperformed the individual ones. However, when it comes to real-time, large-scale, complex, and high-dimensional data such as images, conventional ML algorithms fall short.

**Table 1:** Summary of the work done in literature for eye disease diagnosis.

Study	Purpose	Method Used	Result
[6]	Key features and DR prediction	Naive Bayes (NB), Sequential Minimal Optimization (SMO), logistic regression (LR), Stochastic Gradient Descent (SGD), bagging classifier, J48 classifier, decision tree (DT), and random forest (RF)	LR achieved the highest accuracy of 75%
[7]	DR classification and identification of most discriminative features	Linear Discriminant Analysis (LDA), Support Vector Machine (SVM), K-Nearest Neighbor (KNN), RF, Ranger Random Forest (RRF)	RRF achieved the highest accuracy of 86%
[8]	An ensemble-based ML model against individual algorithms for predicting DR	RF, DT, Adaboost, KNN, LR	Ensemble model outperformed individual models and achieved an accuracy of 82%
[9]	A two-stage hybrid approach for automated DR classification using segmentation and feature extraction to detect retinal biomarkers	U-Net for segmentation, hybrid CNN-SVD model, Inception-V3 based transfer learning	Achieved the highest accuracy on EyePACS dataset of 97.92%
[10]	A method for accurate haemorrhage detection using modified contrast enhancement	Modified pre-trained CNN, convolutional sparse image decomposition, multi-logistic regression for feature selection	Achieved the average accuracy of 97.71%
[11]	A DL-based framework for DR screening, optimized for resource-constrained edge devices	MobileNetV2, MobileNetV3, NasNetMobile, EfficientNetV2B0, InceptionV3	MobileNetV2 achieved the highest accuracy of 98.4% accuracy
[12]	An intelligent model to classify all five stages of DR using fundus images	Vision Transformer (ViT)	Achieved an accuracy of 96.35% on class 1
[13]	A Vision Transformer-based method to recognize DR grades and replace CNNs with attention mechanisms	ViT	Achieved accuracy of 91.4%
[14]	Transformer-based models to capture crucial features in retinal images for understanding DR severity and improve model performance	ViT, class attention in image transformers (CaiT), bidirectional encoder representation for image transformers (BEiT) and data efficient image transformers (DeiT)	94.63% accuracy
[15]	A ViT-based method for automated DR severity classification and its comparison with CNN-based mdoels	ViT, ResNET101, InceptionV3	ViT achieved the highest accuracy of 87.41%
[16]	Comparative analysis of ViTs and CNNs for referable DR detection	Developed 5 CNN models (VGG19, ResNet50, InceptionV3, DenseNet201, EfficientNetV2S) and 4 ViT models ($VAN_{small}$, $CrossViT_{small}$, $ViT_{small}$, $SWIN_{tiny}$)	SWIN transformer achieved the highest AUC of 95.7%
[17]	Comparative analysis of attention-based networks (Transformers), CNNs, and multi-layer perceptrons (MLPs) for DR detection	EfficientNet, ResNet, Swin-Transformer, ViT, and MLP-Mixer	The Swin-Transformer achieved the highest accuracy of 86.4%
[18]	Comparative analysis of ViT and CNN models for glaucomatous optic neuropathy (GON) detection	ViT and ResNET50	ViT models outperformed CNN models in recall (sensitivity) by an average of 0.14 across six datasets

On the other hand, DL-based models designed to handle complex, high dimensional data and are highly scalable and have better domain knowledge and that’s why they outperformed ML models. Among DL models, CNN models were found to be exceptionally good when it comes to image classification or detections tasks. In study [9], Bilal et al. proposed 2 stage methodology for DR classification. To enhance the quality of images, data augmentation and data preprocessing technique was used in the first step. And in second step, a symmetric hybrid model was used. The proposed methodology achieved an accuracy of 97.92%. In [10], another multistage methodology was proposed for haemorrhage detection, which is one of the earliest symptoms of DR. The proposed methodology achieved an accuracy of 97.72%. In study [11], five pre-trained CNN architectures were compared after fine-tuning on the EyePACs dataset. The study concluded that MobileNetV2 outperformed the other architectures. Further hyperparameter tuning of MobileNetV2 resulted in an increased accuracy of 98.4%. Despite showing good results, CNN-based models face certain challenges, such as multi-dimensional and multi-modal data fusion, data augmentation for real-world variability, privacy-preserving model training, bias in AI systems to name a few, as discussed in [19], with limited generalizability being one of them.

To address the shortcomings of CNN-based models, multiple transformer-based approaches for automated DR detection and classification were adopted [12–18]. Gu et al., in study [12] proposed a classification model for detecting all five stages of DR. The model consisted of two blocks, feature extraction block (FEB) and grading prediction block (GPB) and the transformer was used in the FEB for capturing detailed features. The comparison showed that their proposed model achieved competitive results to those found in the literature. Wu et al. [13] proposed a Vision Transformer (ViT) based model for DR grade recognition. The proposed work achieved an accuracy of 91.4%. In [14], Adak et al. employed an ensemble transformer model consisting of ViT, CaiT, BEiT and DeiT, for detecting the severity of DR. The model achieved 94.63% accuracy. Some works, including [15–18], compare different DL-based models, specifically transformer- and CNN-based models, in terms of performance.

Despite these advancements, the adaptation of such techniques to real-world scenarios remains limited. A major reason for this gap is the inability of models to maintain performance in dynamic, real-world conditions. Many studies overlook the root causes of poor generalizability, and there is currently no standardized framework to evaluate model robustness against diverse data biases or to measure the impact of individual biases on performance.

To analyze the inherent generalizability of any model, it is first necessary to understand the underlying causes of bias. Biases can arise at various stages of the ML pipeline and significantly affect generalization. Therefore, identifying and characterizing these biases, along with their correlation to model performance, is a critical step.

In this study, we begin by conducting an in-depth analysis of bias categorization and their correlations. We then propose a framework to assess the inherent generalizability of models in the presence of these biases. Finally, we present a comparative evaluation of a simple CNN-based model and a Transformer-based model using widely adopted performance metrics such as accuracy, sensitivity, and specificity. The primary objective is to compare the inherent generalizability of a simple CNN-based model with that of a Transformer-based model, excluding attention-based CNN variants. Furthermore, we introduce the BAG index, a performance metric developed in this work. This metric provides insight into the overall generalizability of a model as well as the contribution of each bias to performance degradation.

## Materials and methods

**Fig 4:**
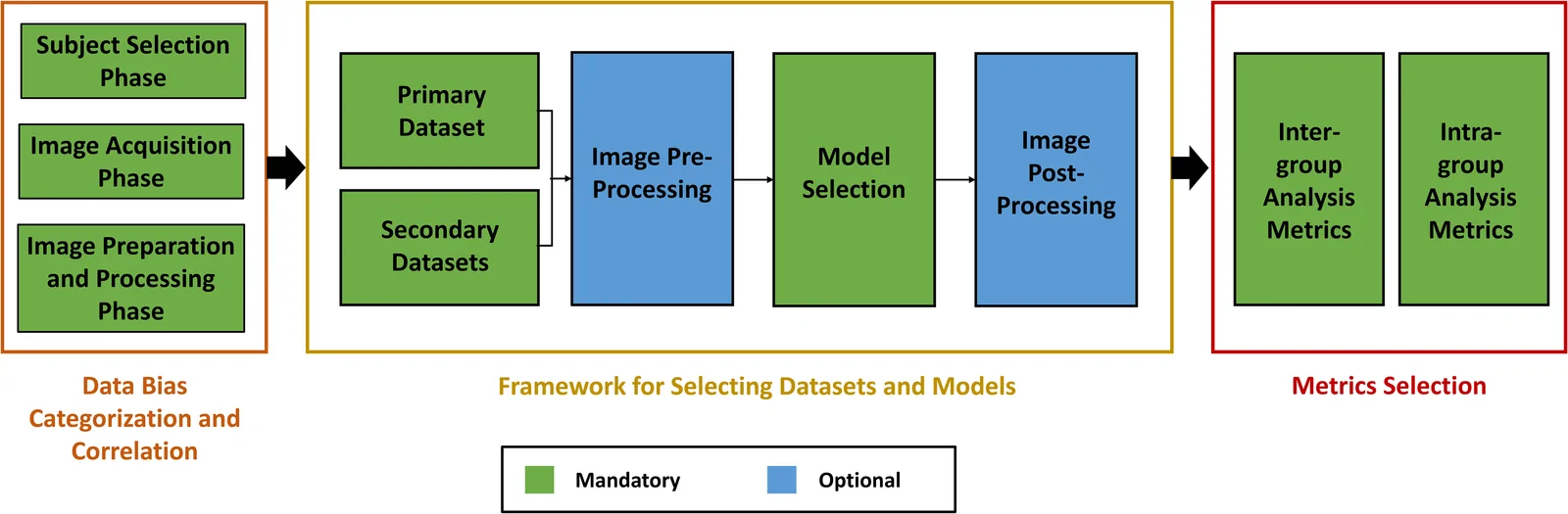
The proposed comprehensive framework for generalizability analysis of an AI model.

In this study, we propose a framework for generalizability analysis designed as a sequential pipeline consisting of three interconnected blocks: (i) systematic categorization of dataset biases, (ii) controlled selection of datasets and model architectures, and (iii) metrics selection for both inter-group and intra-group analysis. An overview of the framework is illustrated in Fig 4.

We then adopted this framework to evaluate and compare the inherent generalizability analysis of 2 models for DR screening. Further details of all blocks of the framework are given in the following subsections.

### Data bias categorization and correlation

This subsection corresponds to the first block of the proposed framework. For generalizability, it is crucial to first understand its root causes. We conducted an in-depth analysis to identify key factors that contribute to poor AI model performance on different diagnostic datasets for the detection of DR. Biases can be introduced at multiple stages of the dataset creation process, including subject selection, image acquisition, and image preparation and processing. Ultimately, these biases lead to reduced generalizability, limiting the model’s ability to perform reliably across diverse datasets.

**Fig 5:**
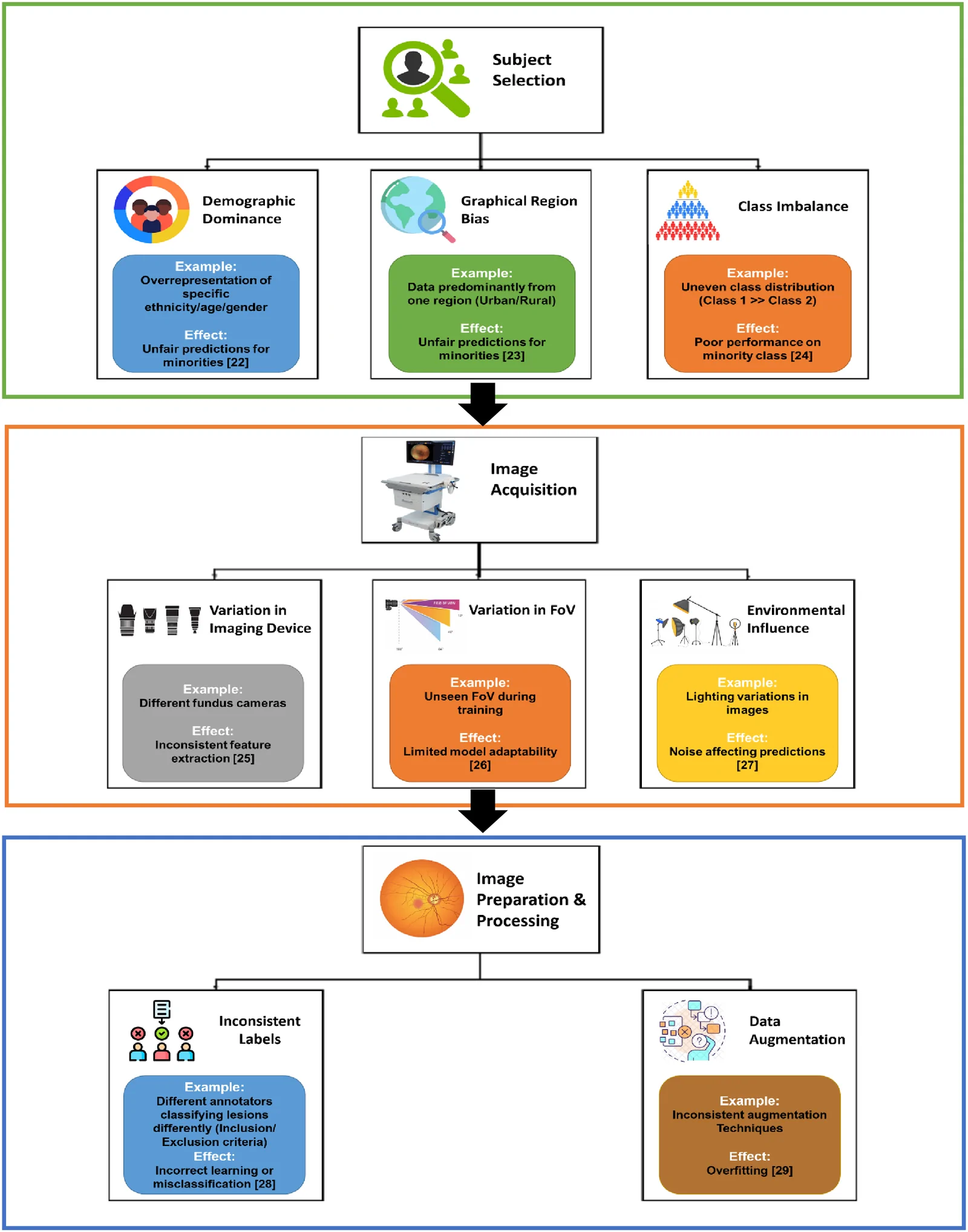
The summary of the bias’s sources, examples and effects in all three stages.

The possibility of introduction of different biases, their examples and effects on the performance of AI model, at any of the previously mentioned 3 stages, as illustrated in Fig 5, are discussed in detail in the following subsections.

#### Subject selection.

In the subject selection phase for the screening of DR, there are multiple factors related to the patient that can result in the introduction of bias in the dataset. The first factor could be the demographics of the patients that include their age, gender, and ethnicity. Normally when a dataset is created, it mostly covers the population of the same race patients such as EyePACS include patients from the USA, APTOS19 and IDRiD from India, Messidor from France, etc. When an AI-based model is trained on only one of these dataset, there is a possibility that it will not perform that well when tested on the dataset of another population. This happens because the anatomical features, such as the retinal vessels, of patients differ from one race to another. There is a difference in the geometry characteristics of the retina vessel between different races as discussed in [20].

Similarly, the model trained on any dataset that has an over-representation of a specific gender or age might under-perform on underrepresented group. The second factor could be the geographical region in which the data collection screening is performed [21]. For instance, datasets dominated by images from urban populations may not generalize well to rural populations, where limited access to prior care and specialized eyecare facilities often results in different disease presentation patterns. A further critical issue is the imbalanced nature of the dataset [22]. This imbalance typically arises from unequal representation of the five stages of diabetic retinopathy (DR). If the dataset contains disproportionately more images from certain stages (e.g., mild DR) and fewer from others (e.g., proliferative DR), the trained AI model will be biased toward the majority classes and struggle to accurately identify the underrepresented stages. Therefore, ensuring balanced class representation across all stages of DR is essential for building robust and clinically reliable models.

#### Image acquisition.

After selecting patients for DR screening, the next step involves capturing the retina. Biases can also be introduced at this stage, primarily due to three factors. First, the difference in the specifications of the devices used for image acquisition [23]. There are multiple publicly available datasets for DR screening and almost each involves images captured from different devices, for example, the Messidor2 dataset was captured using Topcon TRC NW6, whereas, the IDRiD dataset was captured using Kowa VX-10. The use of different capturing devices may introduce variations in the quality of captured images, so the model trained on a dataset captured from Topcon device might not perform well on images captured from another device such as Kowa, Canon, ZEISS, etc.

Second, we look at the variations in the field of views (FoVs) of the capturing devices. FoV represents how much area of the patient’s retina is captured during image acquisition. Different cameras have different FoVs, such as the Canon CF-60 which is used to capture the high resolution fundus (HRF) dataset having an FoV of $60^{o}$, on the other hand, the Topcon TRC NW6 which is used to capture the Messidor2 dataset having an FoV of $45^{o}$. If the model trained on HRF dataset that has the higher FoV is tested on the Messirdor2 dataset with lower FoV, the accuracy of the model will decrease. This difference in the result due to the change in FoV is also discussed in the study [24]. The third factor could be the different environmental factors, such as the difference in lighting and illumination conditions when capturing the image. This can lead to changes in brightness, sharpness and the overall entropy of images, which in turn affects the performance of the model [25].

#### Image preparation and processing.

Once the images have been captured, the next step is to label them and apply any necessary processing technique. Labeling the images is a very critical task, as even a slight inaccuracy can lead to major consequences. There is a high possibility of introduction of bias during labeling [26], as the same image can be labeled differently by different annotators based on their experience and the inclusion exclusion criteria they use. Some annotators prefer not to include images of the retina that has undergone laser surgery, others find no problem with including such images. This can lead to incorrect learning and misclassifications.

The last factor that can contribute to data bias is data augmentation [27]. Although not all datasets involve this stage, in some cases it is crucial to add diversity in datasets. However, when using any augmentation technique, one should make sure that it is absolutely necessary and represents the real-world scenario. In some cases, creating augmented images of the same dataset can lead to model overfitting, meaning it is so well trained on one type of data that when it is tested on other type of data, the performance greatly degrades.

### Framework for selecting datasets and models

**Fig 6:**
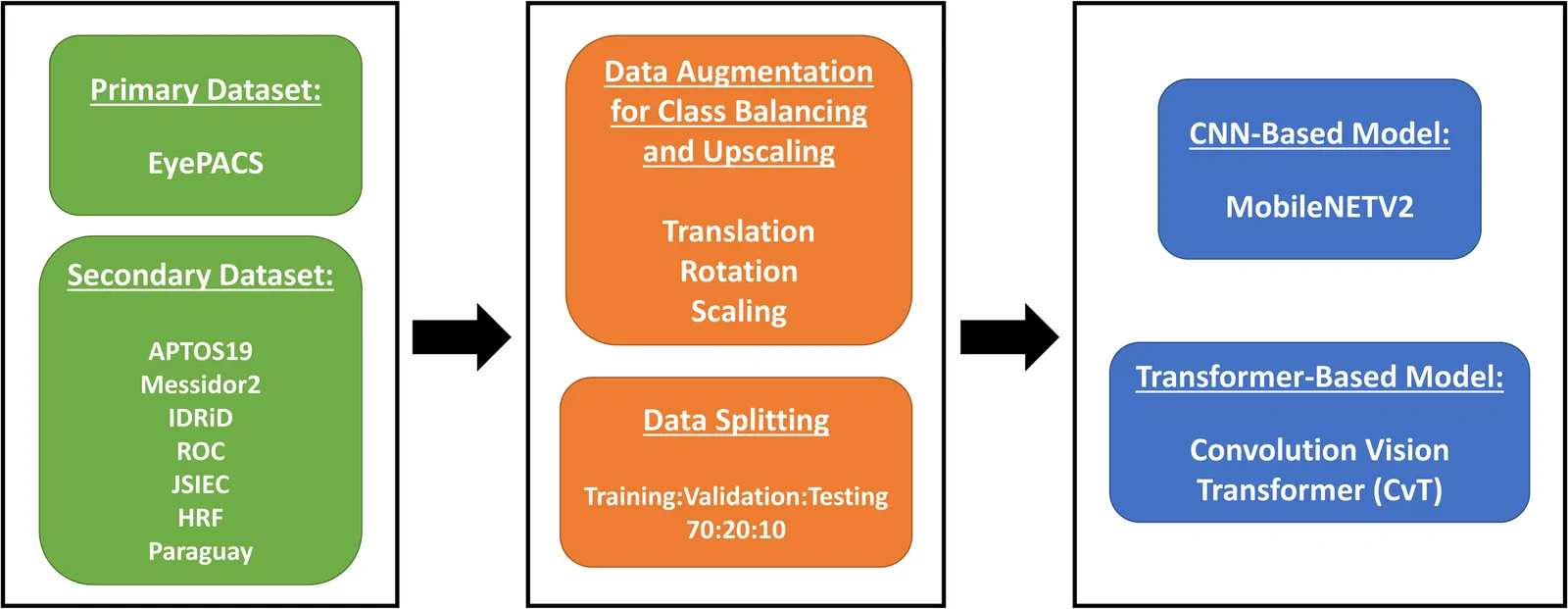
The adaptation of the proposed framework for selecting datasets and models for generalizability analysis of DR screening solutions.

This subsection implements the second block of the framework. The complete framework is divided into several processes, including selection of datasets, image preprocessing, and model selection (CNN-based and transformer-based model), as illustrated in Fig 6.

#### Datasets.

For this study, we have two types of datasets. The first is the primary dataset used for both training and testing, while the second is the secondary dataset, used solely for testing model performance and generalizability. For the primary dataset, we used data from one of the publicly available retinal fundus image datasets, EyePACS [28]. The purpose of choosing this dataset was to have a higher number of labeled images and a higher quality of the images. The images were captured using fundus cameras with $45^{\circ }$ FoV. Originally, the dataset contained 88,702 images, but we worked with only 35,126 labeled images, which are divided into 5 classes of DR namely no DR, mild DR, moderate DR, severe DR and proliferative DR (PDR) [11]. The total number of images found in the dataset against each of these 5 classes is presented in Table 2.

**Table 2:** Original Distribution of EyePACS dataset across 5 stages.

DR Stages	Number of Images
Stage 0 (No DR)	25,802
Stage 1 (Mild DR)	2,438
Stage 2 (Moderate DR)	5,288
Stage 3 (Severe DR)	872
Stage 4 (Proliferative DR)	708

The secondary dataset consists of nine other publicly available datasets. These datasets include Asia Pacific Tele-Ophthalmology Society (APTOS) 19 [29], which was developed for APTOS19 Blindness detection competition, Messidor2 [30], which was developed by the Messidor project to test approaches for DR grading and automatic lesion segmentation, Indian Diabetic Retinopathy Image Dataset (IDRiD) [31], that was the first database representative of an Indian population, Retinopathy Online Challenge (ROC) [32], this microaneurysms database was developed as a part of an online competition held by three well-known researchers working in the field of retinal image processing, Joint Shantou International Eye Centre (JSIEC) [33], these images were taken in JSEIC, Shantou city, Guangdong province, China, High-Resolution Fundus (HRF) [34], the database is provided by the Pattern Recognition Lab, the Department of Ophthalmology, Friedrich-Alexander University Erlangen-Nuremberg (Germany), and the Brno University of Technology, Faculty of Electrical Engineering and Communication, Department of Biomedical Engineering, Brno (Czech Republic), Paraguay [35], acquired at the Department of Ophthalmology of the Hospital de Clínicas, Facultad de Ciencias Médicas, Universidad Nacional de Asunción, Paraguay, Brazilian Multilabel Ophthalmological Dataset (BRSET) [36], a multi-ethnic dataset representing a Latin American population with images obtained from PhysioNet following completion of required CITI training and data use agreement, and Diabetic Retinopathy Detection Dataset (DDR) [37], a large-scale Chinese dataset comprising images collected across multiple independent clinical sites using three distinct imaging devices, providing substantial multi-device acquisition diversity.

The choice of datasets for the secondary dataset was quite critical. We selected these datasets on the basis of their authenticity, the use of different imaging devices with different FoVs, and most importantly, the different population they represent. This was done to ensure that each of these datasets is different from another, which will eventually lead to the introduction of data biases and make our proposed generalizability analysis framework accurate and authentic. These datasets are functions of their specific parameters including demographic data, size, environmental factors (lightning), imagine device factors (sharpness, entropy etc) and the FoV of the camera. In general form, we can write it as shown in Eq (1)


$$\begin{array}{c} D_{i}=\{DG_{i},S_{i},EF_{i},IDF_{i},FoV_{i},\cdots \} \end{array}$$ (1)

where

1≤i≤9

DG = Demographics

S = Size

EF = Environmental Factors

IDF = Imaging Device Factors

FoV = Field of View of the camera

Since these parameters are different for each dataset, we can conclude this difference as expressed in Eq (2). This implies that all datasets differ from each other.


$$\begin{array}{c} \forall \;i,j\in \{1,2,\ldots ,9\},\;i\neq j\Rightarrow D_{i}\neq D_{j} \end{array}$$ (2)

The details of all datasets used in this study are discussed in Table 3, where the first is the primary dataset and the rest are secondary datasets.

**Table 3:** Summary of primary and secondary datasets used for generalizability analysis.

Dataset Name	Number of Images	Population Included	Device Used	FoV	URL
EyePACS	35,126	American	Varies	$45^{o}$	[28]
APTOS19	3662	Indian	Varies	Not listed	[29]
Messidor2	1748	French	3CCD camera on a Topcon TRC NW6 nonmydriatic	$45^{o}$	[30]
IDRiD	516	Indian	Kowa VX-10 alpha digital Fundus	$50^{o}$	[31]
ROC	100	Dutch	Canon CR5–45NM, TopconNW 100 and NW 200	$45^{o}$	[32]
JSIEC	1000	Chinese	ZEISS FF450 Plus IR Fundus Camera, and Topcon TRC-50DX Mydriatic Retinal Camera	$35^{o}$ – $50^{o}$	[33]
HRF	45	German	Mydriatic fundus camera CANON CF–60 UVi equipped with CANON EOS–20D digital camera	$60^{o}$	[34]
Paraguay	757	Paraguayans	Visucam 500, Zeiss brand	Not listed	[35]
BRSET	16,266	Brazilian (multi-ethnic)	Canon CR2, Nikon NF5050	$45^{o}$	[36]
DDR	13,673	Chinese (multi-site)	Canon, Zeiss Visucam 500, Kowa VX-10	$45^{o}$	[37]

Of all the images available in these datasets, we have selected the most suitable for DR detection and then applied geometric data augmentation techniques such as rotation, translation, and scaling to increase the size and variability of these datasets. The total number of images used for testing, in each of the nine dataset discussed, is given in Table 4.

**Table 4:** Total number of images in nine datasets used for testing.

Dataset	Number of Images
APTOS19	600
Messidor2	600
IDRiD	600
ROC	260
JSIEC	180
HRF	270
Paraguay	757
BRSET	800
DDR	800

**Fig 7:**
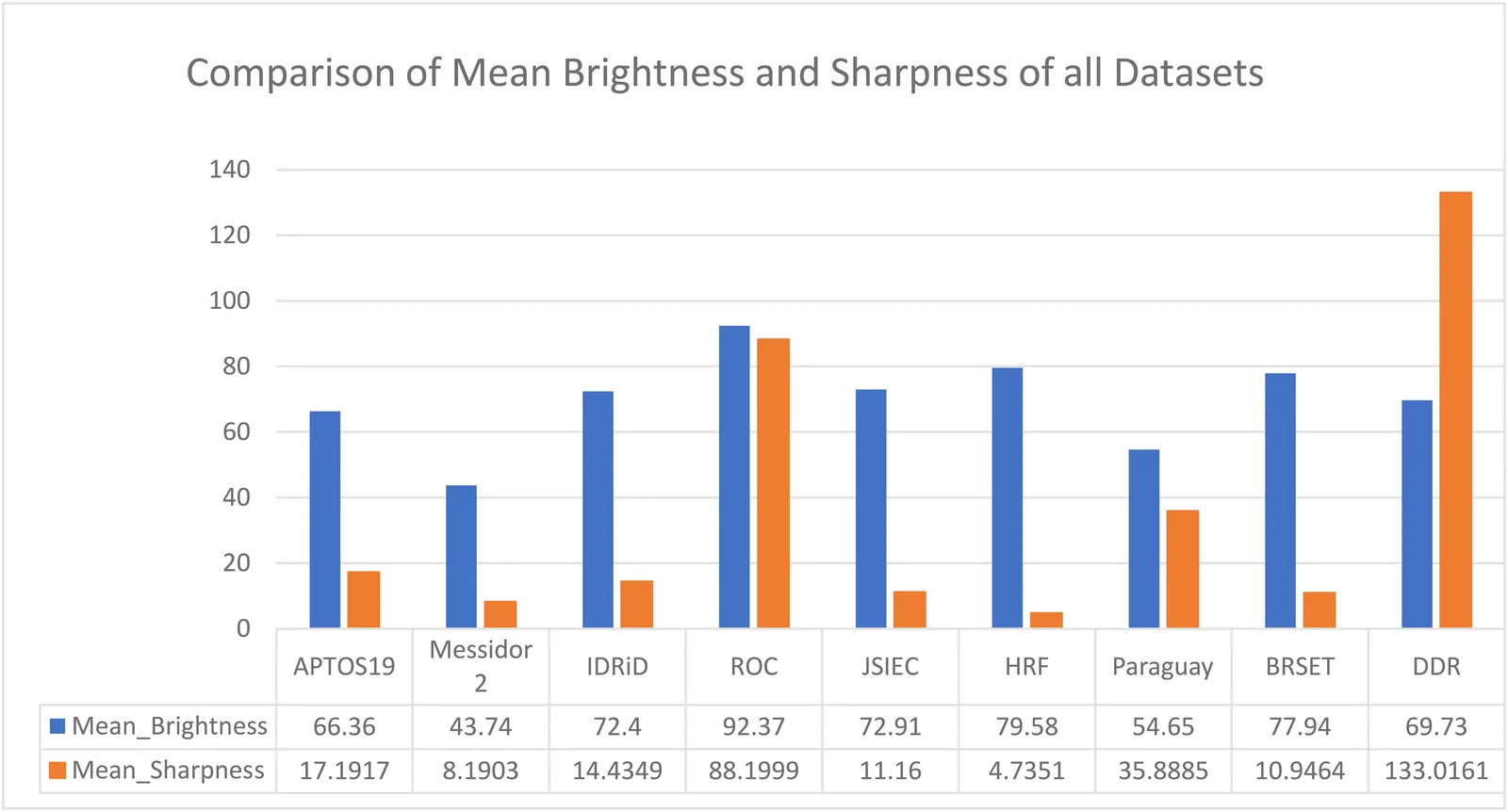
Comparison of mean brightness and sharpness of all secondary datasets.

To further highlight the differences across the secondary datasets, we calculated the mean brightness, which reflects variations in lighting, and the mean sharpness, which indicates image focus or blur. The results, presented in Fig 7, clearly demonstrate the variation in overall image quality among the datasets in terms of brightness and sharpness. These differences are critical for validating their suitability in generalizability analysis.

#### Image processing.

**Fig 8:**
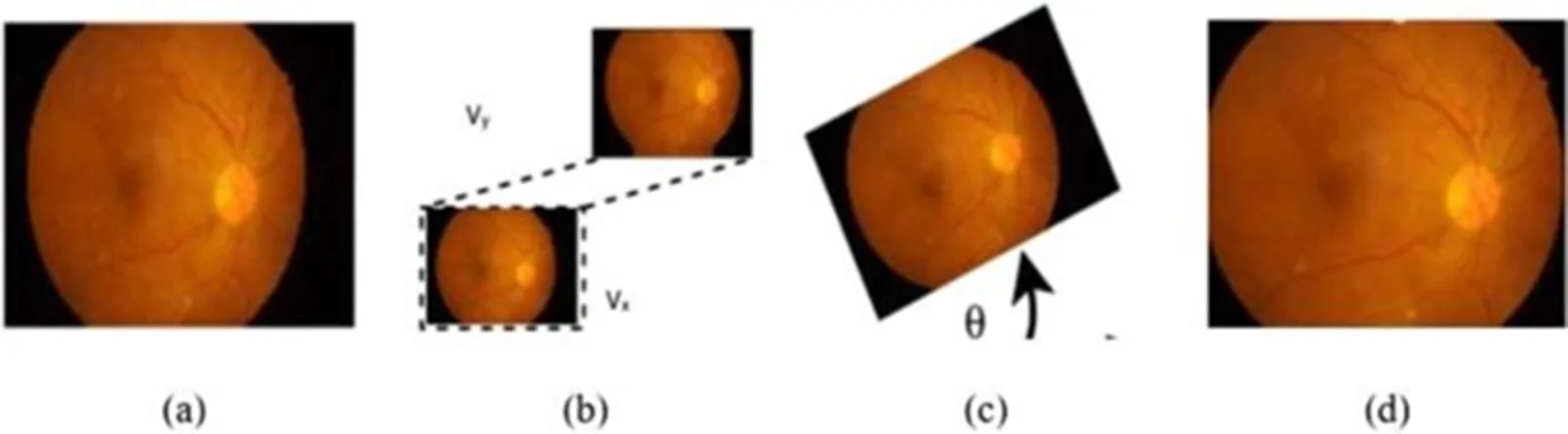
Geometric augmentation techniques utilized in our work: (a) Original image (b) Image Translation with respect to Vx and Vy axis (c) Image Rotation with respect to angle(theta) (d) Image Scaling by zooming in.

**Fig 9:**
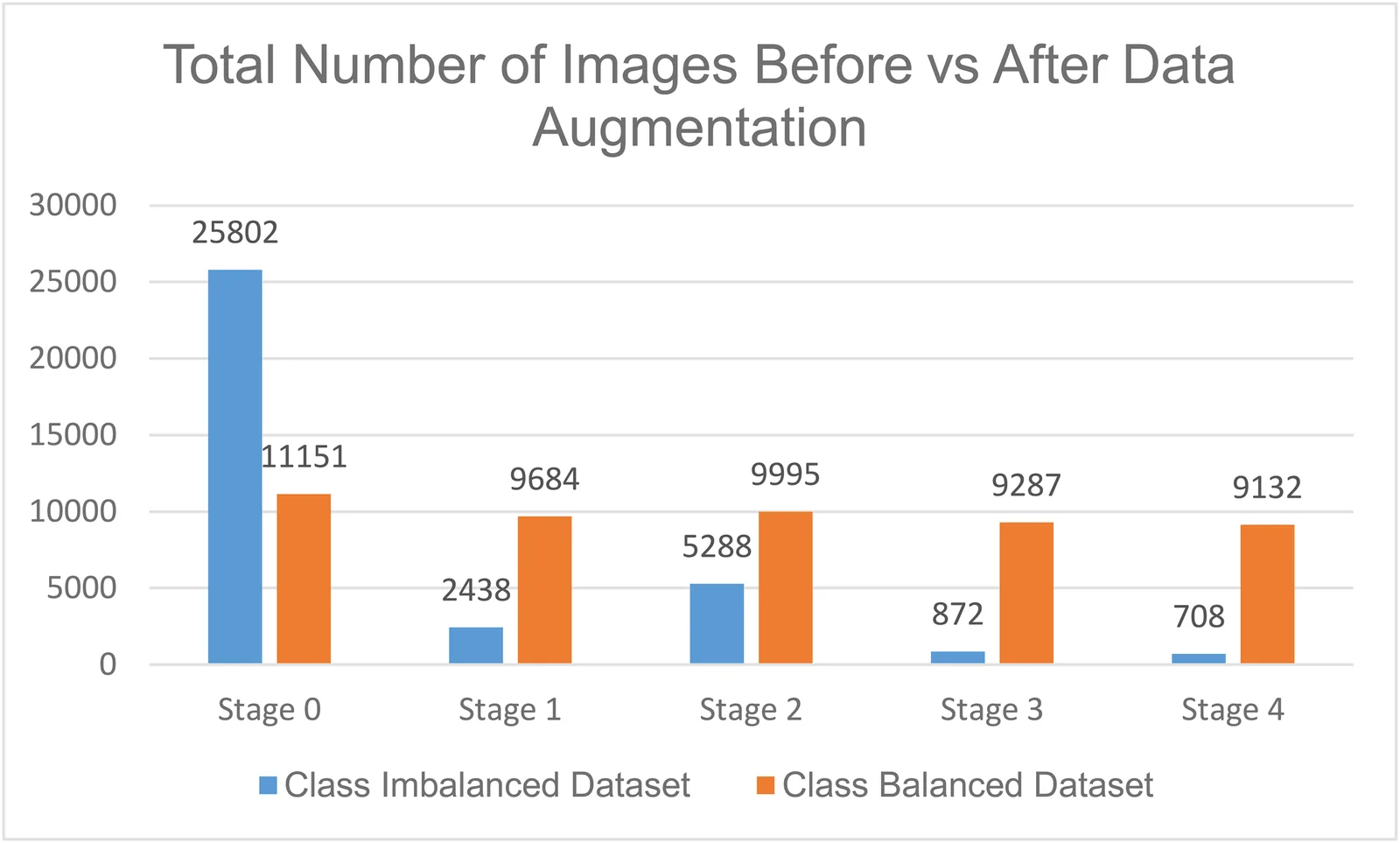
The uniform distribution of EyePACS dataset across 5 stages after applying augmentation techniques.

1. **Data augmentation:** The class imbalance issue leads to over-fitting, a problem arises when a model is trained too well on some specific class or type of data, and it results in low testing accuracy. In EyePACS, the imbalanced ratio(IR) for each DR stage lies between 10.5 to 36.4 with respect to the majority class. To address the class imbalance issue in the primary dataset, geometric image augmentation techniques, such as translation, rotation, and scaling are used. For data augmentation, a random setup was used with a displacement of 10 pixels along the x and y axes, denoted as Vx and Vy for translation, ± 20 degrees for rotation, and 0.2 zoom range for image rescaling. Compared to the original image, the output images produced by translation, rotation, and rescaling are displayed in Fig 8. The original imbalanced dataset for multiclass DR stages is transformed into a new dataset that shows the balanced number of images for each DR stage. The majority class (stage 0) images are downscaled with a ratio of 0.43, while the other minority classes are upscaled with varying scaling ratios to balance the image count for all stages shown in Fig 9.
2. **Data splitting:** After addressing the class imbalance issue, the images in stage 0 and stage 1 are grouped together to represent a class called non-referable DR (Nrdr) and the remaining 3 stages are grouped together into another class called referable DR (Rdr). This step is performed to convert the multiclass classification into binary class classification. For the non-referable class, we selected a total of 3000 images, 1500 from each of the 2 stages, and for referable, we selected 1000 images from each of the 3 stages, which made up to 3000 images as well. This results in a balanced dataset of 6000 images, which was divided into training, validation, and testing sets with a ratio 70% (4200 images), 20% (1200 images), and 10% (600 images) respectively. To guarantee equal representation of each class in each partition, stratified splitting was used. By preserving a 50%/50% ratio of referable to non-referable cases, each split avoided sampling bias and guaranteed representative performance estimate across test, validation, and training sets [38].

#### Selection of models.

Several CNN-based models are available for DR screening, including MobileNetV2 [39], MobileNetV3 [40], NASNetMobile [41], EfficientNetV2B0 [42], and InceptionV3 [43]. Among these, MobileNetV2 was selected due to its lightweight architecture, which is beneficial for edge deployment. In addition to being lightweight, it provides superior performance compared to other CNN-based architectures, as proposed in [11].

Selecting an appropriate transformer-based model for comparison was challenging, as most studies used ViT, first introduced in [44]. Although ViT marked a breakthrough in applying transformers to CV, its performance remained average compared to traditional CNN-based models, possibly due to the absence of certain inherent CNN properties.

Other transformer-based models, such as CvT [45], Swin Transformer [46], BEiT [47], and DeiT [48], have also been explored for DR screening. Among these, CvT outperformed all others by integrating CNN properties with ViT, improving both performance and efficiency. By combining convolutional structures with the self-attention mechanism of transformers, CvT bridges the gap between CNNs and transformers, making it the preferred choice for our comparative analysis. Further details of these architectures are provided below.

**Fig 10:**
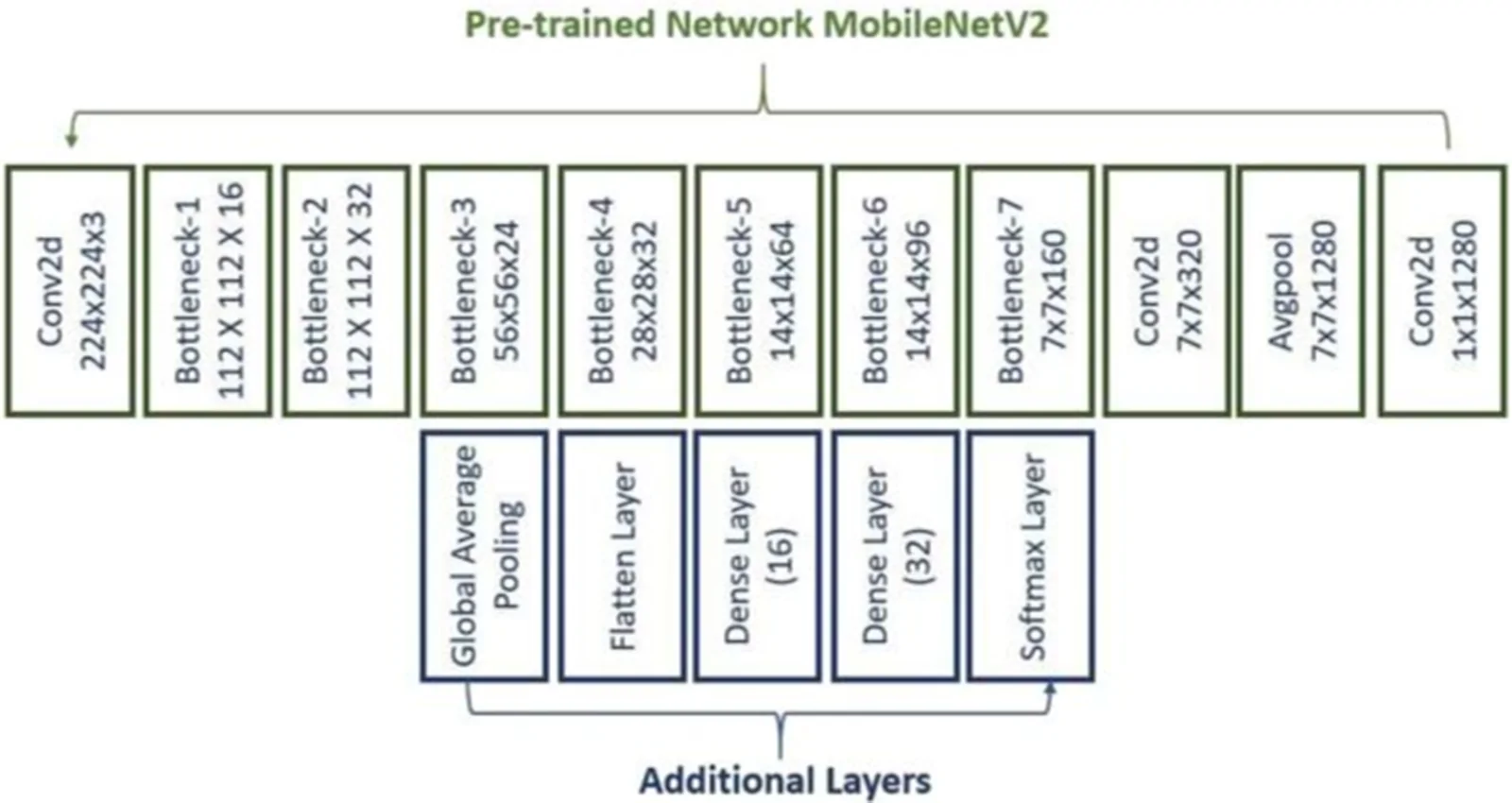
Customized MobileNetV2 architecture with pretrained network layers and 5 additional layers for training.

**Fig 11:**
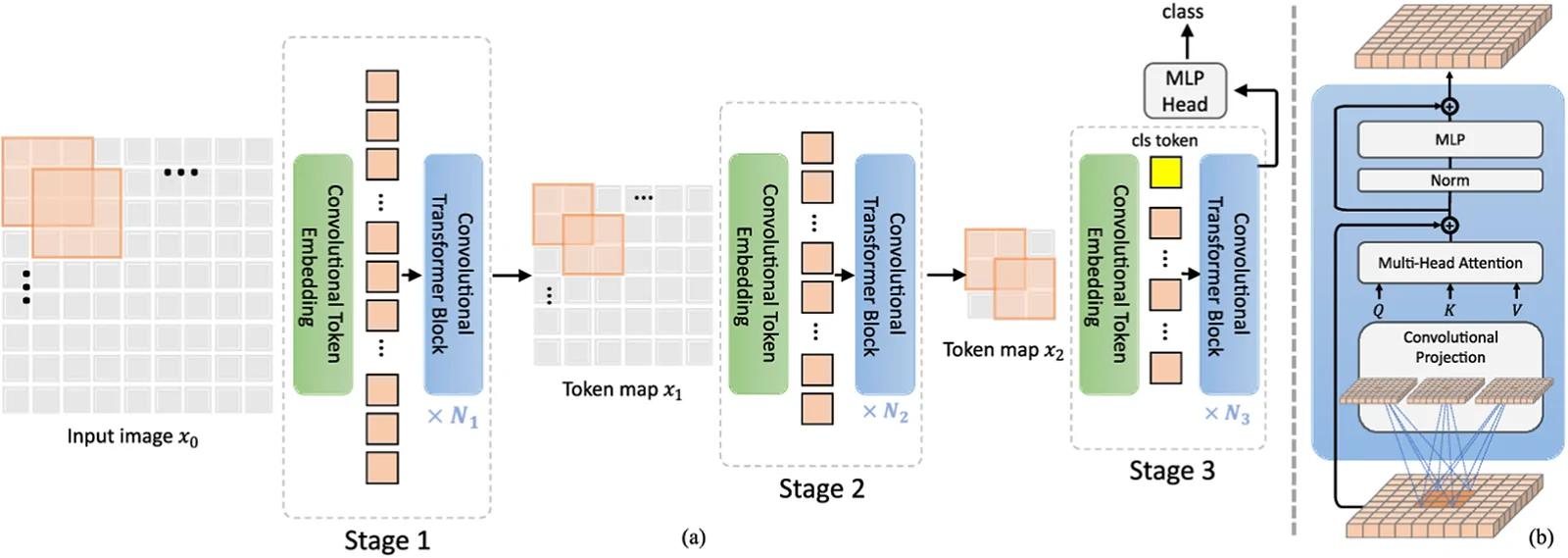
CvT Architecture (a) Overall Architecture (b) Detailed Convolutional Transformer Block [45].

1. **MobileNetV2 architecture:** The MobileNetV2 architecture [39], a pre-trained model on the ImageNet dataset, consists of bottleneck residual blocks followed by a (1x1) convolutional layer, global average pooling layer, and a classification layer. Three convolutional layers; an expansion layer (1x1 convolutional layer) to increase the number of channels, a depth wise convolutional layer for filter operations, and a projection layer (1x1 convolutional layer) to project high-dimensional output into a lower dimension, make up MobileNetV2’s main block which is shown in Table 5. This main block is followed by a batch normalization layer and a ReLU6 activation function. For spatial convolutions, (3x3) kernels are employed. In our proposed work, MobileNetV2 is integrated with 5 more additional layers, including an average pooling layer, a flatten layer, two dense layers with ReLU, and a SoftMax layer for classification. The complete architecture is illustrated in Fig 10. The customized architecture consists of approximately 2.3 million trainable parameters and has a computational cost of 2.368 × 10^3^ GFLOPs. The model size is 14 MB, with an inference time of 0.199 seconds per image on a CPU and 0.169 seconds on a GPU.
2. **CvT architecture:** The architecture of CvT as illustrated in Fig 11. [45], comprised of 3 stages. Each of these stages has two main parts, Convolutional Token Embedding (CTE) and Convolutional Transformer Block (CTB). The CTE module converts the input image into sequence of tokens and an additional normalization layer then reduces the number of tokens generated. When provided with a 2D shaped output token map from a previous stage $x_{i-1}\in {\mathbb{R}}^{H_{i-1}\times W_{i-1}\times C_{i-1}}$ as the input to stage i, it learns a function f(.) to map previous token map to new token map. The height and width of this new token map are given in Eq (3) and (4). It is then flattened and normalized for input into the CTB.

$$\begin{array}{c} H_{i}=\frac{H_{i-1}+2p-s}{s-o}+1 \end{array}$$ (3)

$$\begin{array}{c} W_{i}=\frac{W_{i-1}+2p-s}{s-o}+1 \end{array}$$ (4)

**Table 5:** Bottleneck residual block transforming from k to k’ channels, with stride s and expansion factor t.

Input	Operator	Output
h x w x k	1x1 conv2d, ReLU6	h x w x tk
h x w x tk	3x3 dwise s = s, ReLU6	h/s x w/s x tk
h/s x w/s x tk	Linear 1x1 conv2d	h/s x w/s x tk’

Where H and W are height and width, calculated using p padding and stride of (s – o). The CTB employs Convolutional Projection, a depth-wise separable convolution, for capturing local and global representations. The use of this type of convolution rather than using linear projections used in standard transformers makes it efficient as it achieves the same result but with less computational power. Lastly, at the last stage, a classification token is added and then a Multilayer Perceptron (MLP) head is used to predict the class of the final output. The CvT architecture consists of approximately 31.54 million trainable parameters and has a computational cost of 24.9 GFLOPs. The model size is 119 MB, with an inference time of 0.683 seconds per image on a CPU and 0.294 seconds on a GPU.

### Metrics selection for generalizability analysis

This subsection defines the evaluation strategy used in the final block of the framework. To evaluate the MobileNetV2 and CvT models on primary and secondary datasets, comprehensive performance profiling is essential. In this study, we conducted both intra-group and inter-group generalizability analyses of the models. For the intra-group analysis, we used performance measures such as accuracy, sensitivity, specificity, precision, F1-score, and area under the receiver operating characteristic curve (AUC-ROC) value. For the inter-group analysis, we used BAG index. All of these performance metrics depend upon 4 values, i.e., True Positive (TP), True Negative (TN), False Positive (FP), False Negative (FN). The description of all these 4 values is provided in Table 6.

**Table 6:** Description of values that are used in the formulae of different performance metrics.

Values	Description
TP	Total number of positive cases that are labelled correctly
TN	Total number of negative cases that are labelled correctly
FP	Total number of positive cases that are labelled incorrectly
FN	Total number of negative cases that are labelled incorrectly

- **Accuracy** The proportion of accurate predictions the model makes over all guesses is its accuracy. Mathematically, it can be represented as given in Eq (5).

$$\begin{array}{c} Accuracy=\frac{TP+TN}{TP+TN+FP+FN} \end{array}$$ (5)

- **Sensitivity** It is also called recall and it is a measure of proportion of positive cases that is predicted correctly as given in Eq (6).

$$\begin{array}{c} Sensitivity=\frac{TP}{TP+FN} \end{array}$$ (6)

- **Specificity** It is a measure of proportion of negative cases that is predicted correctly as given in Eq (7).

$$\begin{array}{c} Specificity=\frac{TN}{TN+FP} \end{array}$$ (7)

- **Precision** It is a ratio of true positive cases to all the positive predictions made as given in Eq (8).

$$\begin{array}{c} Precision=\frac{TP}{TP+FP} \end{array}$$ (8)

- **F1-score** It is the harmonic mean of precision and recall as given in Eq (9).

$$\begin{array}{c} F1-score=2\times \frac{Precision\times Recall}{Precision+Recall} \end{array}$$ (9)

- **AUC-ROC** It is the measure of the model’s ability to distinguish between classes and is plotted with x axis containing false positive rate and y axis containing true positive rate (recall).

Although these metrics provide valuable insights into a model’s predictive performance on a single dataset (intra-group analysis), they do not capture the model’s consistency across datasets with varying biases or domain characteristics (inter-group analysis). To address this, we propose BAG index as a more accurate measure of inter-group generalizability.

**BAG index:** By explicitly considering a set of bias factors B, including mean brightness, sharpness, entropy, dataset size, FoV difference, and population score, BAG index evaluates a model’s relative performance on a secondary dataset compared to its performance on a primary dataset. The computation of BAG index involves multiple steps, outlined as follows:

1. **Normalized bias magnitude for each bias *b*:** The normalized bias difference $D_{b}$ quantifies the magnitude of a specific bias *b* between the primary and secondary datasets. It is computed using formula given in Eq 10:

$$\begin{array}{c} D_{b}=\frac{\left|x_{\text{secondary},b}-x_{\text{primary},b}\right|}{\max(x_{b})-\min(x_{b})} \end{array}$$ (10)

where *x*_secondary,*b*_ and *x*_primary,*b*_ represent the value of bias *b* in the secondary and primary datasets, respectively. The denominator, $\max(x_{b})-\min(x_{b})$, normalizes the bias difference across all datasets, ensuring $D_{b}\in [0,1]$. This prevents distortion due to varying scales or units of different biases and avoids dominance by biases with inherently larger numeric ranges.

2. **Normalized performance drop:** The normalized performance drop captures the relative degradation of model performance when evaluated on a secondary dataset compared to the primary dataset. It is computed using Eq 11:

$$\begin{array}{c} \Delta P=\max\left(0,\frac{P_{\text{primary}}-P_{\text{secondary}}}{P_{\text{primary}}}\right) \end{array}$$ (11)

where *P*_primary_ and *P*_secondary_ denote the model’s performance (accuracy, in this study) on the primary and secondary datasets, respectively. The max(0,·) ensures that performance improvements ($P_{\text{secondary}}>P_{\text{primary}}$) do not yield negative values, thus emphasizing degraded performance as an indicator of poor generalizability.

3. **Bias-adjusted drop (BAD) per bias:** The Bias-Adjusted Drop modulates the raw performance drop by incorporating the normalized bias magnitude. It is calculated by using the formula given in Eq 12

$$\begin{array}{c} BAD_{b}=\Delta P*\left(1+D_{b}\right) \end{array}$$ (12)

Here, $D_{b}$ is the normalized bias magnitude for bias *b*. Adding 1 in the denominator ensures that even when there is no difference in bias ($D_{b}=0$), performance drops are still penalized. This formulation penalizes drops more strongly in “easy” low-bias scenarios, while allowing greater tolerance in “hard” high-bias scenarios.

4. **Aggregation into BAG index:** Finally, the BAG index aggregates the bias-adjusted drops across all considered biases. The formula is given in Eq 13

$$\begin{array}{c} BAGindex=1-\frac{\sum_{b}w_{b}\cdot BAD_{b}}{\sum_{b}w_{b}(1+D_{b})} \end{array}$$ (13)

where $w_{b}$ is the weight assigned to bias *b*, allowing customization of its importance. The numerator accumulates the weighted bias-adjusted drops, while the denominator normalizes this sum with respect to the total weighted bias magnitudes plus baseline. Subtracting from 1 transforms the value into a generalizability index:

BAGindex→1: good generalization (no performance drop despite biases).
BAGindex→0: poor generalization (large performance drop relative to biases).

The BAG index uses accuracy as the primary performance metric because the datasets used in this study are explicitly balanced (50% referable and 50% non-referable DR cases). Under such conditions, accuracy becomes equivalent to the average per-class recall, providing an unbiased and interpretable measure of overall classification performance. This avoids the asymmetric weighting effects that may arise in metrics such as precision or F1-score under different class distributions.

Importantly, the BAG formulation is inherently metric-agnostic, as the performance drop term ΔP can be computed using any bounded performance metric without altering the structure or validity of the framework. To further guide the applicability of the framework, Table 7 includes a summary of recommended metrics for ΔP computation under different evaluation scenarios. All selected metrics are bounded within [0,1], where higher values indicate better performance.

**Table 7:** Recommended performance metrics for BAG index computation under different evaluation scenarios.

Evaluation Scenario	Recommended Metric for ΔP	Rationale
Balanced binary classification (this study)	Accuracy	Equivalent to average per-class recall under balanced datasets; directly interpretable overall correctness
Imbalanced binary classification	AUC-ROC	Threshold-independent measure of separability; robust to class imbalance
High clinical cost of missed detection (e.g., DR screening)	Sensitivity	Prioritizes correct identification of positive cases (referable DR)
Need to minimize false positive rate (e.g., screening confirmation stages)	Specificity	Measures ability to correctly identify negative cases, reducing false alarms and overtreatment
High cost of false positives (e.g., unnecessary referrals, confirmatory testing burden)	Precision	Ensures that predicted positive cases are truly positive, reducing unnecessary clinical workload
Balanced trade-off between false positives and false negatives	F1-score or Balanced Accuracy	Captures trade-off between precision and recall; Balanced Accuracy ensures equal class contribution
Multi-class DR severity grading	Balanced Accuracy	Stable and interpretable metric that remains bounded in [0,1] and avoids chance agreement bias

## Experimental results

Following the framework described in the previous section, this section presents experimental results validating the proposed bias-aware generalizability analysis. We first establish baseline performance on the primary EyePACS dataset for both MobileNetV2 and CvT architectures. We then conduct comprehensive cross-dataset evaluation across nine geographically and technically diverse secondary datasets, employing both traditional metrics and the proposed BAG index to quantify architectural resilience to dataset biases.

### Experimental setup

In order to train, test and evaluate both CNN and transformer-based models, we use the hardware platform containing 32GB RAM,10th generation CPU, along with GPU NVIDIA Quadro RTX 5000 with 16 GB RAM and i7th processor. On the other hand, the software environment consists of Windows 10 OS, and for training the pretrained transformer-based models, we have used PyTorch version 2.1 and other relevant and necessary libraries like numpy and pandas. We have employed transformations such as CenterCrop, Compose, Normalize, RandomHorizontalFlip, RandomResizedCrop, Resize, ToTensor, on the EyePACS dataset used in this study, by using transformers module of torchvision. Moreover, to use the GPU during training, NVIDIA’s Compute Unified Device Architecture (CUDA) Application Programming Interface (API) was used. Seaborn and MLxtend were used to visualize confusion matrices and classification reports, and scikit-learn was used to compute comprehensive performance metrics like accuracy, sensitivity, specificity, precision, F1-score, and AUC-ROC. During inference, Python’s time library was used for measuring the inference time.

### Training and evaluation of MobileNetV2 and CVT on EyePACS

Both MobileNetV2 and CvT have different characteristics, including total number of trainable parameters, model size, FLOP count, etc., and due to this reason, both of them might require a different number of epochs to correctly train on the EyePACS dataset. MobileNetV2 generally requires around 40 epochs to converge, whereas CvT typically requires more than 40 epochs. But for a fair comparison, we trained both models for a maximum of 80 epochs under similar conditions. Although training was allowed to proceed for a fixed number of epochs to visualize convergence behavior, model selection for evaluation was governed by early stopping, with the best validation checkpoint retained. In addition, a ReduceLROnPlateau strategy was employed to adapt the learning rate once validation performance saturated.

For MobileNetV2, the Adam optimizer was used with a learning rate of $1\times 10^{-4}$, $\beta_{1}=0.9$, $\beta_{2}=0.999$, and $\varepsilon =1\times 10^{-7}$. Adam is widely used for medical image fine-tuning due to its adaptive learning rates, which help stabilize training on smaller datasets. A conservative learning rate was chosen to avoid large updates that could disrupt pre-trained features [49,50]. Binary cross-entropy was used as the loss function. Regularization is primarily handled through built-in batch normalization layers, which stabilize training, along with early stopping based on validation performance to prevent overfitting.

For CvT, the AdamW optimizer was used. AdamW applies weight decay directly to the model parameters, which is important for transformer-based models that are prone to overfitting during fine-tuning on small datasets. A lower learning rate of $5\times 10^{-5}$ was used compared to training-from-scratch settings, as is standard practice to preserve pre-trained representations during fine-tuning. In this case, weight decay serves as the main explicit regularization mechanism [51,52].

**Fig 12:**
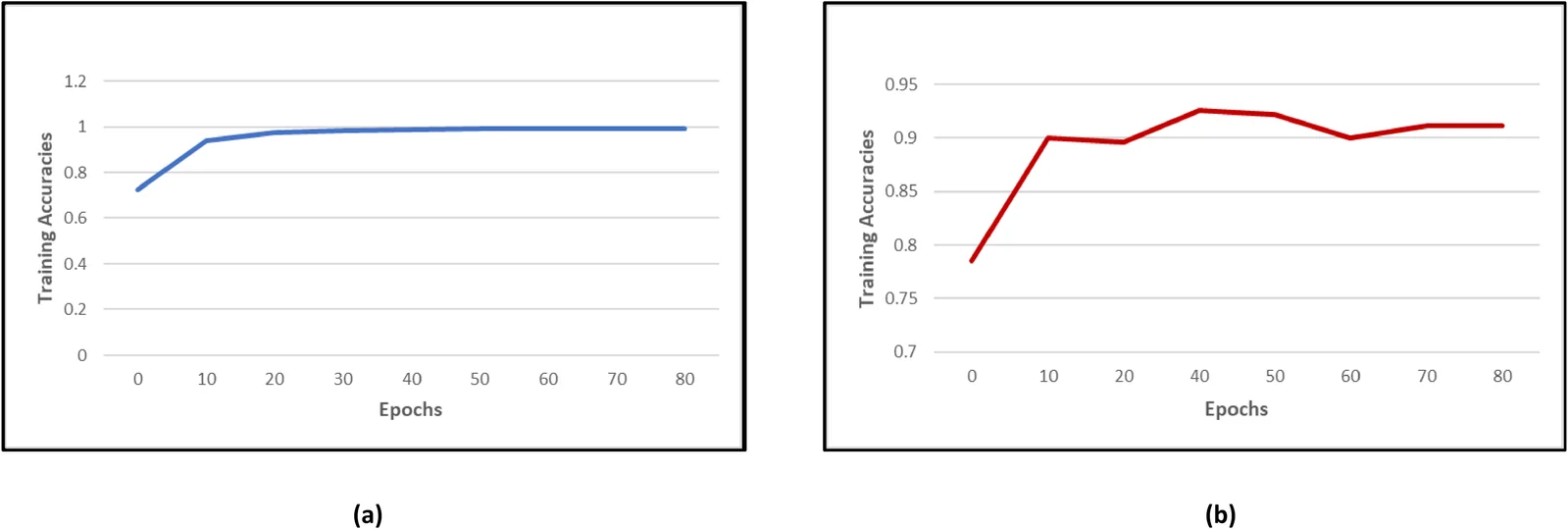
Training accuracies till 80 epochs of (a) MobileNetV2 (b) CvT.

This selection was made on the basis of the training trends observed for both models. As shown in Fig 12, MobileNetV2’s accuracy stabilized after approximately 40 epochs, whereas CvT’s performance plateaued at later epochs, with no significant improvement beyond that point. The flat behavior observed in later epochs reflects convergence rather than overfitting. MobileNetV2 achieved a training accuracy of 0.94, a training loss of 0.15, and a validation loss of 1.180. On the other hand, CvT achieved a training accuracy of 0.95 along with a training loss of 0.2 and a validation loss of 0.12.

Moreover, the evaluation results of the MobileNetV2 and CvT models on the EyePACS dataset are given in Table 8. CvT achieved superior results on the EyePACS dataset. However, achieving good results on one dataset does not necessarily imply that the model will generalize well to unseen datasets; therefore, a detailed generalizability analysis was carried out for both models.

**Table 8:** The summary of performance of MobileNetV2 and CvT on EyePACS dataset.

Performance Metrics	MobileNetV2	CvT
Accuracy	0.855	0.9083
Sensitivity	0.81	0.873
Specificity	0.9	0.943
Precision	0.89	0.9390
F1-score	0.84	0.9050
AUC-ROC	0.85	0.91

### Generalizability analysis

**Fig 13:**
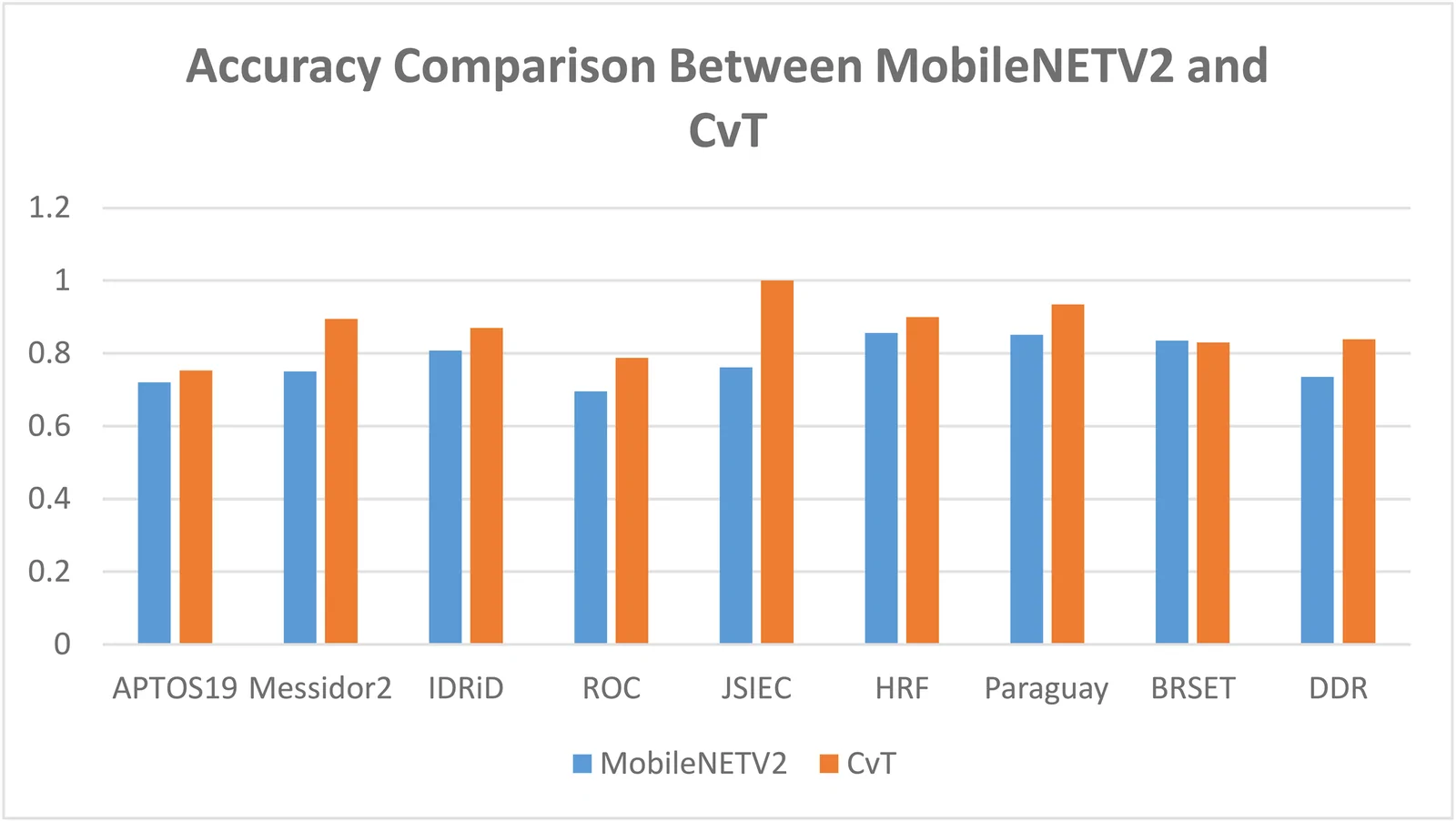
Accuracy comparison between MobileNetV2 and CvT across nine datasets.

**Fig 14:**
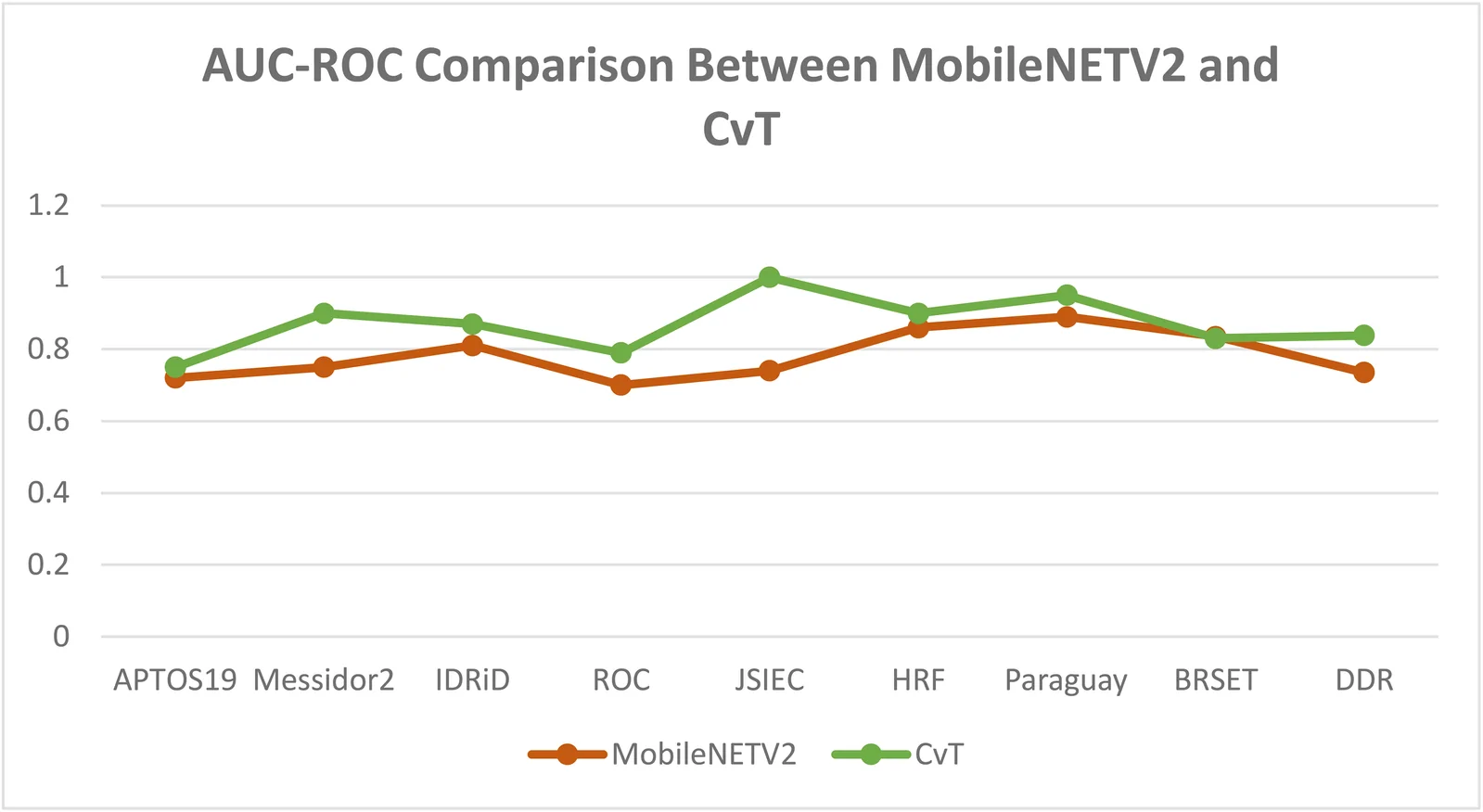
AUC-ROC value comparison between MobileNetV2 and CvT across nine datasets.

For thorough generalizability analysis, both intra- and inter-group analysis were performed using different performance metrics. Table 9 summarizes the performance of MobileNetV2 and CvT across all discussed intra-group analysis measures on the selected datasets. Additionally, Fig 13 provides a comparison of the accuracy of MobileNetV2 and CvT on these datasets, while Fig 14 illustrates the comparison of their AUC-ROC values. The results show that CvT consistently outperforms MobileNetV2 on all nine datasets, both overall and individual, without employing any generalizability enhancement techniques.

**Table 9:** Generalizability Comparison between MobileNetV2 and CvT.

Performance Metrics	MobileNetV2									
	APTOS19	Messidor2	IDRiD	ROC	JSIEC	HRF	Paraguay	BRSET	DDR	Overall Result
**Accuracy**	0.72	0.75	0.808	0.696	0.761	0.856	0.851	0.835	0.735	78%
**Sensitivity**	0.963	0.736	0.943	0.523	0.989	0.71	0.814	0.938	0.795	82.3%
**Specificity**	0.48	0.763	0.673	0.869	0.487	1.0	0.958	0.732	0.675	73.7%
**Precision**	0.649	0.756	0.742	0.8	0.697	1.0	0.982	0.778	0.71	79%
**F1-score**	0.775	0.746	0.831	0.632	0.818	0.831	0.890	0.85	0.75	79%
**AUC-ROC**	0.72	0.75	0.81	0.70	0.74	0.86	0.89	0.835	0.735	78%
	**CvT**									
**Accuracy**	**0.753**	**0.895**	**0.87**	**0.788**	**1.0**	**0.9**	**0.934**	**0.83**	**0.838**	**87%**
**Sensitivity**	1.0	0.933	0.983	0.892	1.0	0.8	0.92	0.97	0.858	93%
**Specificity**	0.506	0.856	0.756	0.684	1.0	1.0	0.97	0.68	0.818	81%
**Precision**	0.669	0.866	0.801	0.738	1.0	1.0	0.99	0.75	0.824	85%
**F1-score**	0.80	0.898	0.883	0.808	1.0	0.889	0.954	0.848	0.841	88%
**AUC-ROC**	0.75	0.90	0.87	0.79	1.0	0.90	0.95	0.83	0.838	87%

Now, to validate these findings through inter-group analysis, the BAG index was calculated. BAG index offers a bias-aware, nuanced assessment of a model’s resilience to domain shifts, in contrast to earlier metrics that solely consider raw accuracy variation. The different biases calculate and incorporated into the BAG index to analyze the dataset differences affecting model generalization, are given in Table 10

**Table 10:** Description of values that are used in the formulae of different performance metrics.

Dataset Bias	Details
Brightness	Overall lighting variations in the dataset relative to the primary dataset
Sharpness	Overall image focus and blur differences compared to the primary dataset
Entropy	Overall image complexity variation relative to the primary dataset
Test Dataset Size	Total number of images in the dataset compared to the size of the primary dataset
FoV	Deviations in the spatial coverage or framing of images relative to the primary dataset
Demographics (Population)	Differences in the population represented by the dataset; 0 if same as primary, else 1

Table 11 presents the BAG index results for CvT and MobileNetV2. Relative to the EyePACS (primary) dataset, MobileNetV2’s BAG index ranges from 1 on HRF (no performance drop) to 0.814 on the ROC dataset (indicating a greater performance drop influenced by dataset biases). Similarly, CvT achieves BAG index ranging from 0.829 on APTOS19–1 on JSIEC and Paraguay.

**Table 11:** Bias-Adjusted Generalizability (BAG) index of MobileNetV2 and CvT across different datasets.

Dataset	MobileNetV2	CvT
APTOS19	0.8421	0.8293
Messidor2	0.8772	0.9857
IDRiD	0.9450	0.9581
ROC	0.8140	0.8678
JSIEC	0.8901	1.0000
HRF	1.0000	0.9912
Paraguay	0.9953	1.0000
BRSET	0.9766	0.9138
DDR	0.8596	0.9226

**Fig 15:**
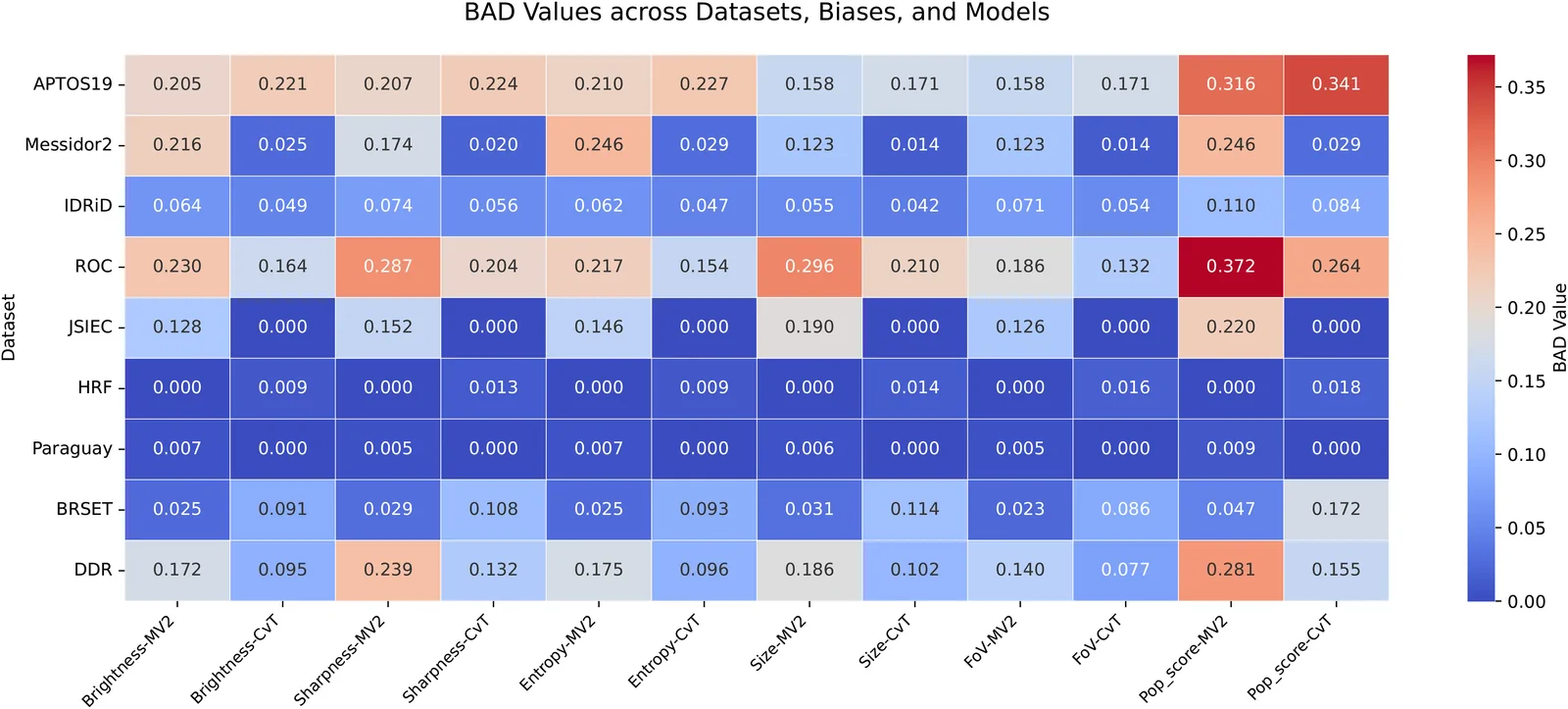
BAD values of MobileNetV2 and CvT across secondary datasets and bias types.

Fig 15 further visualizes the BAD values across all datasets, biases, and models. The heatmap highlights that MobileNetV2 generally records higher BAD values than CvT, particularly for brightness, sharpness, and entropy-related biases, which aligns with its lower BAG index. In contrast, CvT consistently demonstrates reduced BAD values, reflecting its stronger robustness against variations in illumination, image clarity, and acquisition conditions. Notably, high BAD values in APTOS19 and ROC indicate stronger domain shifts affecting generalization, while datasets such as HRF and Paraguay show near-zero BAD values for both models, confirming minimal bias effects. Overall, this comparative BAD analysis complements the BAG index results by illustrating how individual bias types drive differences in generalizability between CNN- and transformer-based models.

To provide further clarity, the following dataset-wise breakdown details the specific biases contributing to performance drops and their correspondence with the BAG index.

- **APTOS19:** Both models experience performance degradation due to high BAD values. Apart from demographic bias, entropy is the major contributor, with values of 0.2099 (MobileNetV2) and 0.2270 (CvT). This reflects the impact of environmental variability and device heterogeneity on generalization. CvT’s slightly higher BAD corresponds with its lower BAG index here.
- **Messidor2:** MobileNetV2 shows higher BAD values across all biases compared to CvT, with entropy being the main factor (0.2456). For CvT, entropy drops to 0.028, suggesting stronger resilience to bias shifts due to its feature extraction capacity. Consequently, CvT achieves a higher BAG index.
- **IDRiD:** Both models record relatively low BAD values, indicating limited bias effects. For MobileNetV2, sharpness is the main challenge (BAD = 0.074), while CvT shows a smaller impact (0.056), corresponding to a higher BAG index of 0.958 versus 0.945.
- **ROC:** MobileNetV2 exhibits the highest BAD values across multiple biases (brightness = 0.230, sharpness = 0.287, entropy = 0.217, FoV = 0.296), reflecting strong domain shifts from multi-device acquisition and FoV variation. CvT, however, consistently reduces BAD values across all biases, achieving a higher BAG index by handling illumination and contrast variability more effectively than CNNs.
- **JSIEC:** For MobileNetV2, the largest performance drop arises from FoV (BAD = 0.190), yielding a BAG index of 0.890. CvT shows no measurable bias effects (BAD = 0) and thus achieves a perfect BAG index of 1, indicating superior generalization.
- **HRF:** MobileNetV2 records zero BAD values across all biases, resulting in a perfect BAG index of 1. CvT, however, experiences a minor performance drop related to dataset size (BAD = 0.0138), yielding a slightly lower BAG index of 0.991.
- **Paraguay:** CvT records zero BAD values and a perfect BAG index of 1, while MobileNetV2 experiences a minor brightness-related drop (BAD = 0.007), giving it a BAG index of 0.995.
- **BRSET:** CvT shows higher BAD values, with size being the main factor (0.114) followed by sharpness (0.108), suggesting transformers may be more sensitive to the multi-ethnic demographic composition that differs substantially from the predominantly American training data. MobileNetV2 shows lower BAD values, with size at only 0.031 and consequently achieves a higher BAG index.
- **DDR:** MobileNetV2 exhibits higher BAD values, with sharpness being the main factor (0.239) followed by size (0.186), reflecting CNNs’ difficulty in handling the multi-device acquisition variability (Canon, Zeiss, Kowa) across multiple clinical sites. CvT shows lower BAD values, with sharpness at 0.132 (nearly half of MobileNetV2’s), demonstrating transformers’ superior ability to adapt to device-induced image quality variations, and consequently achieves a higher BAG index.

The results make it clear that the generalizability profiles of both models across datasets can be explained by their BAD values. CvT consistently minimizes BADs, particularly when handling complex brightness, sharpness, and FoV variations. In contrast, MobileNetV2 is more susceptible to these biases, leading to higher BAD values and lower BAG index scores, except in low-bias datasets where both models perform similarly. The final aggregated BAG index, computed as the average across secondary datasets, are 0.94 for CvT and 0.91 for MobileNetV2. These results further support the notion that transformer-based models are inherently more resilient to dataset biases and thus achieve stronger generalizability.

## Discussion and future directions

This section interprets the experimental findings in the context of dataset bias, architectural design, and practical deployment constraints. First, the relationship between key architectural traits (self-attention, multi-scale feature extraction, dynamic receptive fields) and observed generalization performance is discussed. Second, computational trade-offs and pathways toward hybrid solutions are presented. Finally, explainability, clinical deployment, and ethical considerations are addressed.

As highlighted in the introduction, understanding dataset biases is essential to assessing model generalizability. Our analysis confirms that architectural design significantly influences how models respond to these biases. CvT demonstrates superior performance compared to MobileNetV2 on seven of the nine datasets, showing greater robustness to variations in FoV, entropy, and sharpness. These advantages arise from three distinct transformer-based design features:

- **Self-Attention Mechanism:** CvT uses self-attention layers to capture long-range and local dependencies, helping the model handle bias-induced domain shifts such as varying image focus or complexity by better understanding spatial relationships and global context [53].
- **Multi-Scale Feature Extraction:** By combining transformer blocks with convolutional layers, CvT captures hierarchical features from fine details to high-level abstractions, enhancing generalization across diverse imaging conditions and devices [54].
- **Dynamic Receptive Fields:** Unlike CNNs’ static convolutional kernels, transformers adaptively weigh input tokens, making receptive fields context-dependent and helping mitigate brightness and FoV-related biases [55].

Despite this, transformer-based models have high computational and memory demands, which limit deployment on edge devices compared to lightweight CNNs such as MobileNetV2. This gap can be narrowed by integrating lightweight attention modules such as Convolutional Block Attention Modules (CBAM), Squeeze-and-Excitation (SE) block into MobileNetV2, constructing hybrid CNN-transformer architectures, applying bias-aware data augmentation and domain adaptation, using deformable convolutions for improved FoV adaptability, and substituting Gaussian Error Linear Unit (GELU), for Rectified Linear Unit (ReLU) activations to improve generalization under varied imaging conditions.

While this study evaluates one representative model per architectural family to isolate the effect of design principles under controlled conditions, the framework’s architecture-agnostic nature means these findings serve as a baseline against which more advanced architectures can be systematically benchmarked in future work. Applying it to architectures such as Swin Transformer, DeiT, and emerging vision foundation models would enable systematic analysis of how advances in attention mechanisms, hierarchical feature learning, and large-scale pretraining contribute to bias tolerance and cross-dataset robustness.

### Extending the BAG framework toward stage-wise analysis and dataset size effects

The formulation of DR detection as binary classification is aligned with the primary objective of population-level screening: identifying patients requiring referral versus those who do not. This approach is adopted in clinically validated systems including the FDA-authorized IDx-DR [56] and landmark DL screening studies [57,58]. In practical workflows, this binary referral decision is the primary actionable outcome, with detailed staging performed subsequently during specialist evaluation.

The BAG framework is not limited to binary classification. Since the BAG index is metric-agnostic, the performance term in ΔP can be replaced with any suitable metric. For balanced datasets, accuracy is sufficient. For imbalanced datasets, macro-averaged F1-score is more appropriate. For ordinal class labels subject to inter-annotator variability, Cohen’s Kappa is suitable [59]. Any bounded metric where higher values indicate better performance preserves the consistency and interpretability of the BAG formulation.

To further generalize the framework, a stage-wise formulation for multi-class severity grading is presented as a formal mathematical extension of the BAG index. For stage-wise multi-class analysis, Step 1 remains unchanged as it measures dataset-level bias. In Step 2, the normalized performance drop is computed separately for each stage s∈{0,1,2,3,4} as shown in Eq 14:


$$\begin{array}{c} \Delta P_{s}=\max\left(0,\;\frac{P_{\text{primary},s}-P_{\text{secondary},s}}{P_{\text{primary},s}}\right) \end{array}$$ (14)

In Step 3, the per-stage BAD is computed as given in Eq 15:


$$\begin{array}{c} BAD_{b,s}=\Delta P_{s}\times (1+D_{b}) \end{array}$$ (15)

In Step 4, the stage-wise BAG index is defined using Eq 16:


$$\begin{array}{c} BAG_{s}=1-\frac{\sum_{b}w_{b}\cdot BAD_{b,s}}{\sum_{b}w_{b}(1+D_{b})} \end{array}$$ (16)

The overall BAG index combines all stages using a weighted average is given in Eq 17:


$$\begin{array}{c} BAG_{\text{overall}}=\sum_{s=0}^{S-1}\pi_{s}\cdot BAG_{s} \end{array}$$ (17)

where the stage weights are defined using Eq 18:


$$\begin{array}{c} \pi_{s}=\frac{\min(N_{s,\text{primary}},\;N_{s,\text{secondary}})}{\sum_{s}\min(N_{s,\text{primary}},\;N_{s,\text{secondary}})} \end{array}$$ (18)

The weights $\pi_{s}$ capture the effective shared sample support per stage. Using the minimum operator ensures that a stage’s contribution is limited by whichever dataset represents it less. Since reliable generalizability assessment requires sufficient samples in both datasets. Stages with few shared samples receive lower weights, reducing the influence of statistically unstable estimates, while well-represented stages contribute more strongly to the final score. This prevents sparsely populated stages from disproportionately affecting *BAG*_overall_ while still preserving their contribution in a controlled manner.

Dataset size is also explicitly incorporated as one of six normalized bias factors. In Step 1, the normalized size bias *D*_size_ represents the difference between primary and secondary dataset sizes, which then directly amplifies *BAD* in Step 3. To illustrate this, Table 12 compares two secondary datasets evaluated on MobileNetV2: IDRiD (*n* = 600), which matches the primary test set size, and JSIEC (*n* = 180), the smallest secondary dataset.

**Table 12:** Comparison of BAG index computation steps for a size-matched (IDRiD) and a smaller (JSIEC) secondary dataset relative to the primary dataset (EyePACS), highlighting the role of normalized size bias.

Step	Formula (for only *b* = size)	IDRiD (*n* = 600)	JSIEC (*n* = 180)
**Step 1: Normalized size bias**	$D_{\text{size}}=\frac{\left|x_{\text{secondary},size}-x_{\text{primary},size}\right|}{\max(x_{\text{size}})-\min(x_{\text{size}})}$	$\frac{\left|600-600\right|}{800-180}=0.000\;↔$	$\frac{\left|180-600\right|}{800-180}=0.677\;↑↑$
**Step 2: Normalized performance drop**	$\Delta P=\max\left(0,\frac{P_{\text{primary}}-P_{\text{secondary}}}{P_{\text{primary}}}\right)$	$\frac{\left|0.855-0.808\right|}{0.855}=0.055\;↑$	$\frac{\left|0.855-0.761\right|}{0.855}=0.110\;↑↑$
**Step 3: Bias-adjusted drop (BAD)**	$BAD_{\text{size}}=\Delta P\times (1+D_{\text{size}})$	0.055×1.000=0.055↔	0.110×1.677=0.184↑↑
**Step 4: BAG index (all biases)**	$BAG=1-\frac{BAD_{\text{size}}}{\left(1+D_{\text{size}}\right)}$	0.945↑	0.890↓

Arrow notation: ↑ higher, ↑↑ much higher, ↓ lower, and ↔ equal relative to the IDRiD dataset.

For IDRiD, *D*_size_ = 0, so *BAD* remains equal to ΔP (0.055). For JSIEC, *D*_size_ = 0.677 amplifies ΔP=0.110, yielding a *BAD* value (0.184) nearly three times that of IDRiD. Importantly, *BAG* reflects the combined effect of all bias factors and ΔP rather than penalizing smaller datasets directly, JSIEC’s *BAG* (0.890) is determined by both its bias magnitude and observed performance drop. Findings from smaller datasets such as JSIEC (*n* = 180) and HRF (*n* = 270) are therefore considered alongside results from larger datasets to ensure generalization conclusions reflect consistent trends across all evaluation settings.

### Model explainability analysis

**Fig 16:**
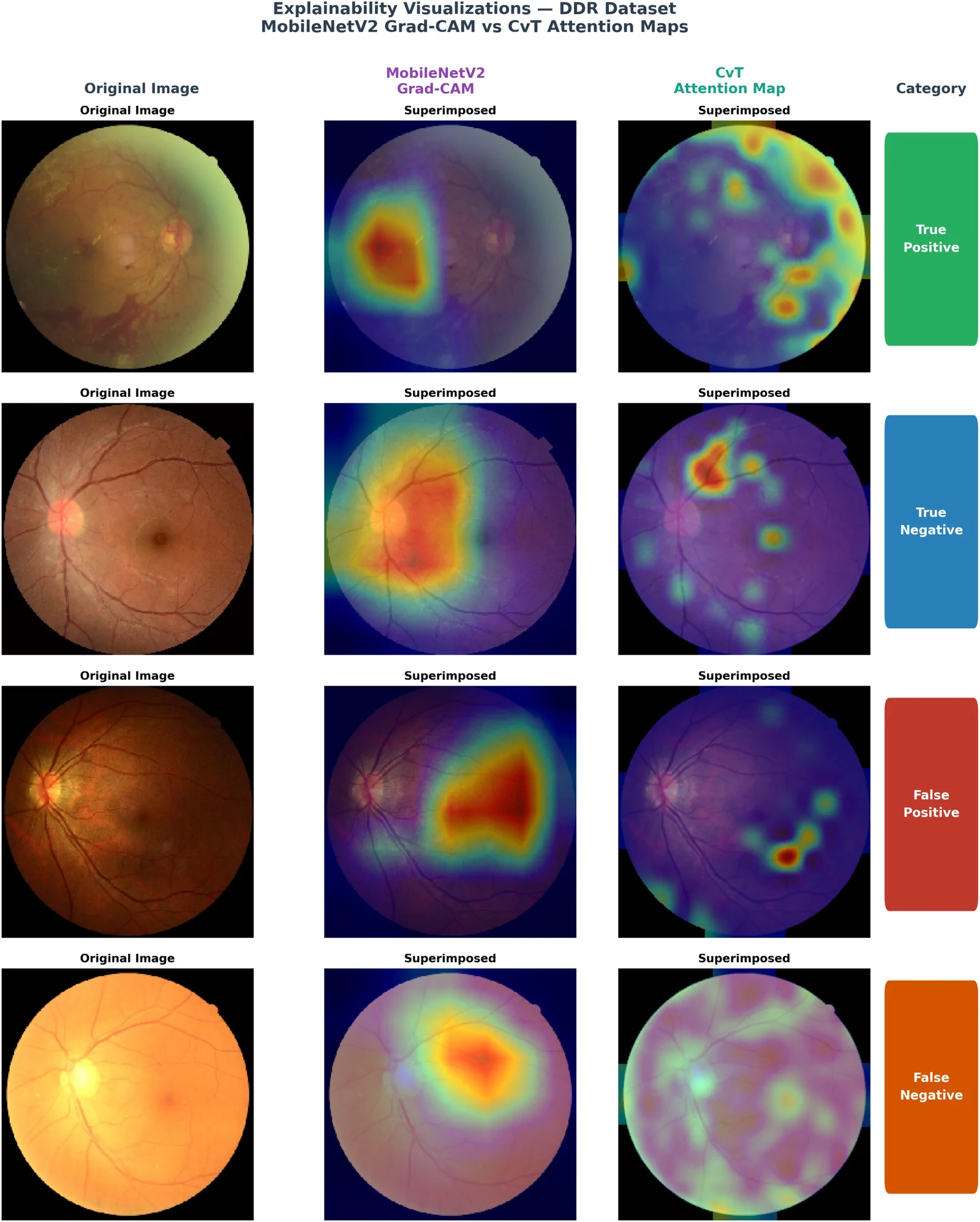
Explainability analysis result of MobileNETV2 and CvT on EyePACS.

**Fig 17:**
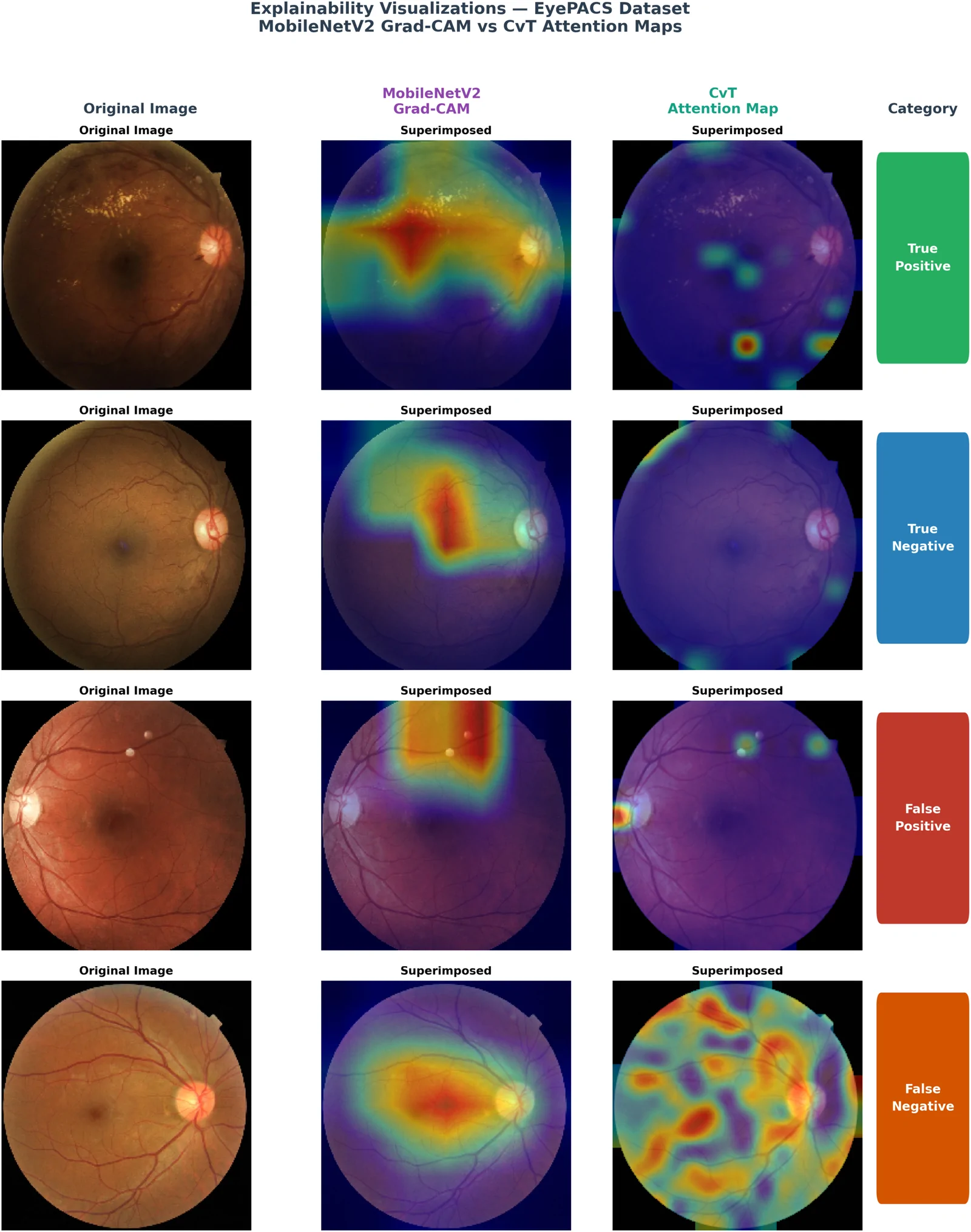
Explainability analysis result of MobileNETV2 and CvT on DDR.

To validate whether the models attend to clinically relevant retinal regions, gradient-based explainability visualizations were generated for both architectures across four prediction categories, TP, TN, FP, and FN, on both the EyePACS and DDR datasets Figs 16 and 17 respectively.

The two architectures produce visually distinct activation patterns by design. MobileNetV2 generates spatially broad, contiguous warm regions consistent with convolutional feature aggregation across neighboring spatial locations. CvT produces sparse, multi-focal activation across discrete patch tokens, consistent with transformer token-level attention that selectively weights individual image patches rather than spatial neighborhoods. This difference in activation character is an architectural property of transformers and has been documented in prior analyses of CNN versus transformer explainability in medical imaging [60,61]. Importantly, CvT’s sparse maps are not a limitation, each hotspot corresponds to a specific patch region identified as discriminative, making them architecturally more precise, while visually less diffuse than MobileNetV2’s broader activations.

- In TP cases, both models focus their activation within the retinal area. The optic disc is not strongly activated, showing that neither model relies on it as a shortcut for classification.
- In TN cases, MobileNetV2 shows weak, scattered activation, while CvT produces almost no activation, indicating a more appropriate response when no important features are present.
- In FP cases, MobileNetV2 shows strong activation in normal regions and makes confident incorrect predictions, suggesting it is influenced by irrelevant features. In contrast, CvT produces weak and sparse activation with prediction scores close to the decision threshold, reflecting better handling of uncertainty.
- In FN cases, MobileNetV2 shows widespread activation despite predicting very low probabilities. CvT maintains distributed activation across multiple regions with near-threshold probabilities, suggesting it still detects subtle signals even though they are not strong enough for correct classification.

Across all categories and datasets, activation maps show that both models focus on retinal regions rather than the background. Similar activation patterns appear in two independently collected datasets that use different imaging devices and patient groups, demonstrating consistency.

### Clinical deployment, ethical considerations, and real-world integration

In the current DR screening workflow, retinal fundus photographs are captured at a primary care clinic or community screening center and reviewed by a trained grader or referred to a specialist, a process that is time-consuming and heavily dependent on specialist availability, particularly in LMICs where the ophthalmologist-to-patient ratio is low. An AI-based screening system can directly be integrated into the existing clinical workflow. Retinal images captured by the facility’s existing fundus camera are passed to the AI screening software, which returns a binary referral decision within seconds. This result is surfaced directly within the facility’s electronic health record system as a flagged alert, enabling clinical staff to prioritize high-risk patients for ophthalmologist referral without waiting for manual grading. For low-risk patients with no retinopathy, annual or biennial follow-up screening may be recommended, while those with detected retinopathy require more frequent review at six-month intervals to ensure timely detection of disease progression [62].

Model selection for deployment depends on infrastructure availability. In urban or centralized settings with GPU access and reliable connectivity, CvT (119 MB, 0.683s CPU inference per image) is suitable for cloud-based or server-side deployment. In rural or resource-constrained settings, MobileNetV2 (14 MB, 0.199s CPU inference per image) enables real-time edge deployment on standard edge devices without GPU support.

DL models’ internal decision-making is not directly visible, limiting clinical trust and regulatory acceptance without supporting evidence [63]. Grad-CAM visualizations for MobileNetV2 and gradient-based attention maps for CvT produce heatmap overlays displayed alongside the referral decision, allowing clinicians to verify that predictions are driven by clinically meaningful retinal features rather than imaging artifacts. Both models are intended as decision-support tools, with a qualified clinician remaining in the loop to make final referral decisions. Continuous post-deployment monitoring and periodic revalidation are essential to handle data drift, varying patient conditions, and disease progression as patient demographics and imaging conditions evolve.

Beyond computational and explainability considerations, responsible clinical deployment requires explicit attention to data governance and algorithmic fairness. All datasets used in this study are publicly available, de-identified, and accessed in accordance with applicable data use agreements and data protection regulations [64]. In deployment, images must be transmitted over encrypted channels, stored in access-controlled environments with full audit logging, and processed solely for screening without retention of personally identifiable information [65]. Access within the clinical interface must be role-based, and anonymized performance logs can be accumulated over time to support periodic model revalidation on locally representative data while maintaining privacy compliance [66].

Algorithmic fairness is addressed by design through the BAG framework, which quantifies and incorporates demographic bias as an explicit bias factor, enabling practitioners to identify where performance degrades across populations before deployment rather than post hoc [67]. Training on a predominantly American population nonetheless introduces demographic bias, as retinal vascular characteristics and disease presentation vary across racial and ethnic groups [20]. Future implementations should incorporate demographically diverse training data, subgroup-level performance monitoring, and fairness-aware learning strategies [68]. The explainability visualizations further support fairness by enabling clinicians to verify that model decisions are driven by pathological features rather than demographic or imaging artifacts.

## Conclusion

This study presents a systematic framework for evaluating the generalizability of deep learning models in diabetic retinopathy screening by explicitly accounting for dataset biases. Through categorization of bias sources and the introduction of the BAG index, we provide a rigorous, bias-aware methodology to assess model robustness across diverse populations and imaging conditions. Comparative analysis between MobileNetV2 and CvT demonstrates that transformer-based architectures exhibit superior resilience to dataset variability, with CvT consistently achieving higher accuracy and BAG scores across multiple external datasets. These results underscore the critical role of architectural design in bias mitigation and highlight self-attention–driven transformers as strong candidates for improving generalizability in clinical AI.

While the computational demands of transformers remain a barrier to deployment in resource-constrained environments, this work opens new pathways for hybrid approaches, where lightweight CNNs can be augmented with transformer-inspired modules to achieve both scalability and robustness. Beyond diabetic retinopathy, the proposed framework offers a transferable methodology for bias-aware evaluation of AI models across other medical imaging domains.

By shifting the focus from narrow performance metrics toward bias-informed generalization, this study provides a foundation for developing AI systems that are not only accurate but also equitable, interpretable, and deployable in diverse healthcare settings. Future research should build on this framework by quantifying the individual contributions of specific biases, extending evaluations to multi-stage DR grading, and exploring efficient hybrid architectures that balance accuracy with clinical feasibility. Ultimately, our findings advance the development of trustworthy AI for global ophthalmic care
